# A Quantitative Risk Assessment Model for *Listeria monocytogenes* in Ready-to-Eat Smoked and Gravad Fish

**DOI:** 10.3390/foods13233831

**Published:** 2024-11-27

**Authors:** Ursula Gonzales-Barron, Régis Pouillot, Taran Skjerdal, Elena Carrasco, Paula Teixeira, Matthew J. Stasiewicz, Akio Hasegawa, Juliana De Oliveira Mota, Laurent Guillier, Vasco Cadavez, Moez Sanaa

**Affiliations:** 1 Centro de Investigação de Montanha (CIMO), Instituto Politécnico de Bragança, Campus de Santa Apolónia, 5300-253 Bragança, Portugal; ubarron@ipb.pt (U.G.-B.); vcadavez@ipb.pt (V.C.); 2 Laboratório para a Sustentabilidade e Tecnologia em Regiões de Montanha, Instituto Politécnico de Bragança, Campus de Santa Apolónia, 5300-253 Bragança, Portugal; 3 Independent Researcher, 18 rue Mohamed Al Ghazi, Rabat 10170, Morocco; 4 Norwegian Veterinary Institute, Section of Bacteriology—Food and GMO, Postbox Sentrum 750, N-0106 Oslo, Norway; taran.skjerdal@vetinst.no; 5 Departamento de Ciencia y Tecnología de los Alimentos, UIC Zoonosis y Enfermedades Emergentes (ENZOEM), Campus de Excelencia Internacional en Agroalimentación (CeiA3), Universidad de Córdoba, Campus Rabanales, Edificio Darwin-Anexo, E-14071 Córdoba, Spain; bt2cajie@uco.es; 6 CBQF—Centro de Biotecnologia e Química Fina—Laboratório Associado, Escola Superior de Biotecnologia, Universidade Católica Portuguesa, Rua Diogo Botelho 1327, 4169-005 Porto, Portugal; pcteixeira@ucp.pt; 7 Department of Food Science and Human Nutrition, University of Illinois at Urbana-Champaign, 905 S Goodwin Ave., Urbana, IL 61801, USA; mstasie@illinois.edu; 8 Nutrition and Food Safety Department, World Health Organization, 1202 Geneva, Switzerland; hasegawaa@who.int (A.H.);; 9 Risk Assessment Department, French Agency for Food, Environmental and Occupational Health & Safety (Anses), 14 rue Pierre et Marie Curie, 94701 Maisons-Alfort, France; laurent.guillier@anses.fr

**Keywords:** brined fish, salted fish, gravlax, listeriosis, exposure assessment, simulation

## Abstract

This study introduces a quantitative risk assessment (QRA) model aimed at evaluating the risk of invasive listeriosis linked to the consumption of ready-to-eat (RTE) smoked and gravad fish. The QRA model, based on published data, simulates the production process from fish harvest through to consumer intake, specifically focusing on smoked brine-injected, smoked dry-salted, and gravad fish. In a reference scenario, model predictions reveal substantial probabilities of lot and pack contamination at the end of processing (38.7% and 8.14% for smoked brined fish, 34.4% and 6.49% for smoked dry-salted fish, and 52.2% and 11.1% for gravad fish), although the concentrations of *L. monocytogenes* are very low, with virtually no packs exceeding 10 CFU/g at the point of sale. The risk of listeriosis for an elderly consumer per serving is also quantified. The lot-level mean risk of listeriosis per serving in the elderly population was 9.751 × 10^−8^ for smoked brined fish, 9.634 × 10^−8^ for smoked dry-salted fish, and 2.086 × 10^−7^ for gravad fish. Risk reduction strategies were then analyzed, indicating that the application of protective cultures and maintaining lower cold storage temperatures significantly mitigate listeriosis risk compared to reducing incoming fish lot contamination. The model also addresses the effectiveness of control measures during processing, such as minimizing cross-contamination. The comprehensive QRA model has been made available as a fully documented qraLm R package. This facilitates its adaptation for risk assessment of other RTE seafood, making it a valuable tool for public health officials to evaluate and manage food safety risks more effectively.

## 1. Introduction

Cold-smoked and gravad fish are products with considerable public health implications in regards to listeriosis since they are not heat-treated, are generally eaten with no prior heating, and have a long shelf life. Over more than a decade, many reports and surveys have highlighted that ready-to-eat (RTE) seafood products are prone to contamination with *L. monocytogenes* [1,2,3,4,5,6]. Data have shown that fish used as raw material can be contaminated with *L. monocytogenes* cells before harvesting, which then multiply previous to smoking or marination when in the processing plant [7]. Implementation of whole genome sequencing (WGS) has revealed that different genotypes dominate in the environment, processing, and in different countries, both in sea and on land [8]. According to the latest European Union (EU) zoonoses report [9], in 2022 RTE fish and fishery products was the food category with the highest occurrence of *L. monocytogenes*, with an overall mean prevalence of 7.1% (N = 9727), varying from 0.0 to 20.0% between Member States (MS).

While qualitative methods provide valuable insights for the risk ranking of pathogens in food chains, a quantitative approach is essential for precisely quantifying the efficacy of control measures, enabling the assessment of risk levels and the mathematical modeling necessary to effectively predict and manage public health outcomes. Quantitative risk assessment (QRA) is a systematic approach used to estimate the risk of infection and illness from exposure to pathogens through various environmental mediums like food, water, or air [10].

Recently, using a “generic quantitative risk assessment” model, EFSA [11] contrasted the risk of listeriosis in the EU elderly population linked to foods products such as RTE fish, pâté, cooked meats, sausages, soft and semi-soft cheeses, and blanched frozen vegetables and determined that gravad fish in normal atmosphere packaging and hot-/cold-smoked fish in reduced-oxygen packaging were the top-most high-risk products. In terms of reported outbreaks, according to EU surveillance data [12], in the period between 2010 and 2020, fish and fish products (namely, crab meat, crustaceans, shellfish, mollusks, smoked fish, and non-specified seafood) caused 23% of the 53 strong-evidence total outbreaks in the EU, whereas, in 2022, EFSA [9] ranked *L. monocytogenes* in fish and fish products as one of the top ten pathogen/food vehicle pairs causing the highest number of deaths in strong-evidence outbreaks in the reporting EU MS. The high share of RTE seafood as a causative agent of listeriosis was also purported by a recent genomic-based epidemiological study [13], which estimated that 27% of 228 listeriosis cases in Germany between 2010 and 2020 were most likely caused by smoked or gravad salmon products.

More recently, ECDC and EFSA [14] investigated a prolonged cross-border outbreak of *L. monocytogenes* ST173 that, between 2017 and 2024, caused 73 cases of listeriosis, including 14 deaths, in Belgium, the Czech Republic, Germany, Finland, the Netherlands, Italy, and the UK. WGS cluster analysis and tracing evidence indicated that the strain spread in Europe originated from a past single source in the fish production chain. Contrary to Europe, where seafood like gravad and smoked fish in certain packaging types pose high risks, the attribution of outbreaks of listeriosis to seafood in the USA appears to be lower [15].

In view of the fact that most of the listeriosis cases occur sporadically [16], it is also pertinent to point out the outcomes of a meta-analysis on case-control studies of sporadic listeriosis [17]. These authors found that, among the RTE food categories, seafood, processed meats, cheese, and composite foods, RTE seafood presented the highest association with sporadic listeriosis, with pooled odd ratios of 10.75 (*p* < 0.001) for non-perinatal populations (immunocompromised and the elderly) and 6.273 (*p* < 0.001) for all susceptible populations (perinatal/non-perinatal, immunocompromised, and the elderly). In order to provide valuable insights on practices and strategies to reduce the current risk of listeriosis associated with RTE seafood products, various QRA models have been developed. A recent critical review on listeriosis QRA models [18] revealed that, although 12 out of the 13 seafood models retrieved (published between 1998 and 2022) investigated the food products specifically considered as high risk—cold-smoked or gravad fish—all of them represented short supply chains only, either from end processing/retail to table [16,19,20,21,22,23,24] or the sole consumption module [25,26,27,28,29,30]. None of the available QRA models included a processing module nor at least a qualitative assessment of the most relevant opportunities of cross-contamination during processing, probably due to the insufficiency of data at the time of their development. In 2022, the Joint FAO/WHO Expert meeting on microbiological risk assessment of *Listeria monocytogenes* in foods recommended the development of a full primary production (harvest and farming) to consumption risk assessment for the risk of *L. monocytogenes* for RTE seafood (hot- and cold-smoked fish and gravad fish) [31].

In light of new data and predictive microbiology models, the objective of this study was to build a QRA model of longer scope for RTE smoked and gravad fish capable of representing the growth, inactivation, and potential cross-contamination from processing to consumption as well as the retarding effect of background microbiota on the development of *L. monocytogenes*.

This study addresses the critical need for an updated quantitative risk assessment model that incorporates the latest data and technological advancements in predictive microbiology. By developing a comprehensive model that evaluates the entire chain of production from primary processing to consumption, this paper aims to provide actionable insights and strategies to significantly reduce the health risks associated with the consumption of RTE smoked and gravad fish.

The model was designed to be able to assess the contribution of the initial contamination of the incoming fish, the contribution of cross-contamination during the filleting of fish and slicing prior to packaging, the effect of the sampling schemes at the end of processing, the effect of time and temperature throughout the logistics of the end-product, the effect of improved practices throughout the supply chain, and the effect of lactic acid bacteria (LAB) cultures added for the biocontrol of *L. monocytogenes*. The model’s structure for both RTE smoked and gravad fish was developed according to the recent Expert Panel recommendation of the Joint FAO/WHO Expert meeting on microbiological risk assessment [31]. The present article aims to describe in detail the QRA model and subsequently illustrate its functionality by reference and what-if scenario analysis.

## 2. Materials and Methods

### 2.1. Exposure Assessment

**Figure 1:**
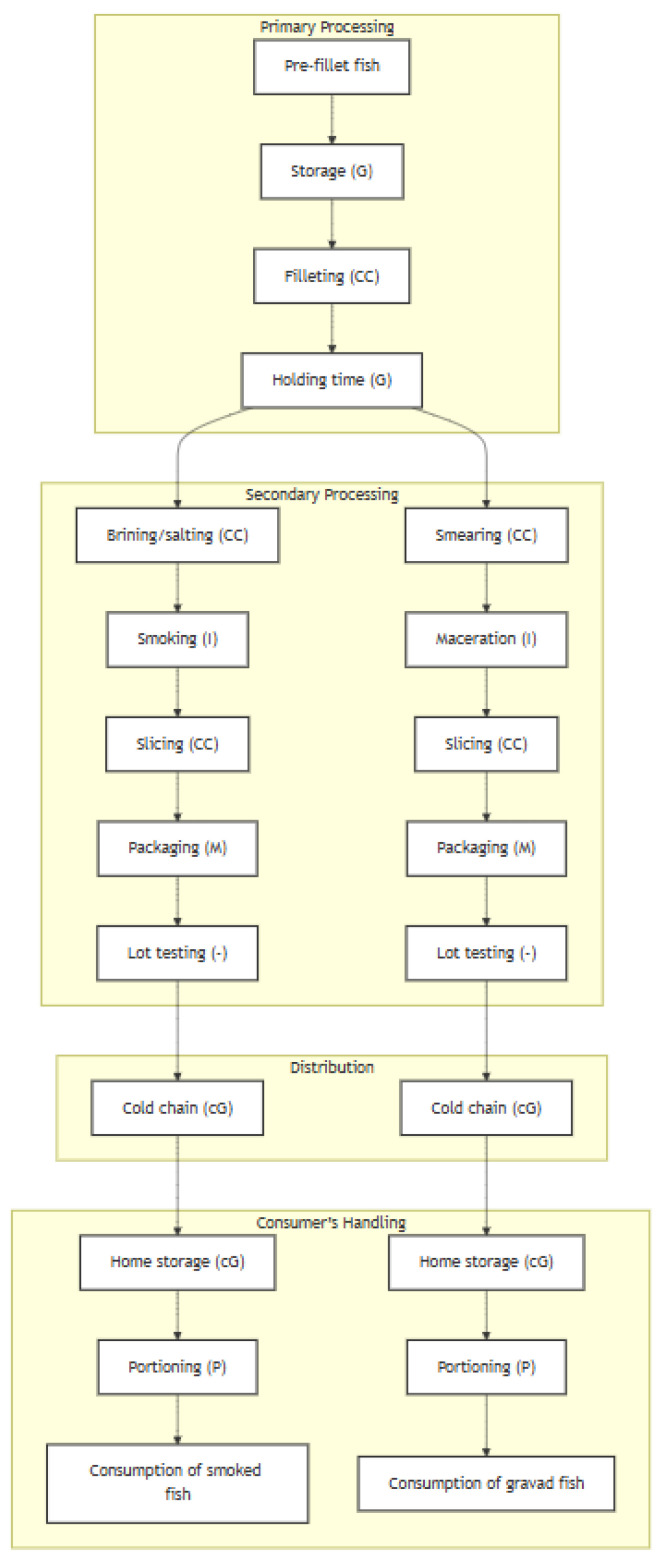
Schematic of the four-module exposure assessment of *L. monocytogenes* in smoked fish (left) and gravad fish (right), with indications of the modelled processes: CC, cross-contamination; G, growth; cG, growth in competition with lactic acid bacteria; M, mixing; I, inactivation; P, partitioning.

The exposure assessment model was developed for two RTE seafood products, smoked fish and gravad fish, and both are presented in this article since they share most of the processing operation units. The processing stages are schematized in Figure 1. The model starts from whole fish units and considers the storage before filleting, which can represent the transport to the primary processing facility or waiting time at the processing facility. After filleting, there is a holding-off time at the facility before preparation, yet it can alternatively correspond to the transport of fish fillets to a secondary processing facility.

At this point, there is a differentiation between smoked fish and gravad fish. For the processing of smoked fish, the stages modelled are salting/brining and smoking/maturation, whereas for the gravad fish the stages are the smearing of fillets with condiments, followed by maceration. The stages that follow are common to both products—the slicing of fillets, packaging, within-lot testing, cold chain distribution (i.e., transport to retail, display at retail, and transport from retail to home), and home storage and portioning—although the parameters feeding the model are product-specific.

Each of the stages shown in Figure 1 was coded as a function stochastically estimating the microbial prevalence and numbers after a process of microbial growth, inactivation, cross-contamination, or partitioning [32], except for within-lot testing, which did not conform to any of the aforementioned processes. Table 1 presents a synthesis of the modules, the sequence of stages and processes they consist of, the assumption and data sources employed, and the corresponding functions programmed in the R software version 4.4.2.

#### 2.1.1. Contaminated Lots of Pre-Fillet Fish

The prevalence of *L. monocytogenes* in pre-processed fish was modeled using published data, assuming that the variability between different lots of fish follows a *Beta* distribution. The model was expressed as

$$s_{j}\sim Binomial\left(n_{j},p_{j}\right)\;p_{j}\sim Beta\left(\alpha ,\beta \right)$$

This distribution is defined by parameters α and β, derived from the survey data listed in Table 2. Each sampling result, from a total of 12 fish lots, is considered a part of a binomial distribution, representing the unobserved prevalence in each lot.

The model employs Bayesian methods with non-informative priors for α and β, set to follow Gamma distributions. After 20,000 iterations in a Markov chain Monte Carlo (MCMC) simulation, the average values for α and β were calculated, with the mean prevalence of *L. monocytogenes* in any given fish lot modeled as Beta (0.8741, 5.880). This represents an average prevalence rate of approximately 14.86%, with a 95% confidence range from 0.037 to 55.41%.

Since, to the best of the authors’ knowledge, the numbers of *L. monocytogenes* in gutted and cleaned fish are very low, mostly below the limit of quantification (10 CFU/g) [7,60], the Poisson assumption was employed to approximate the concentration of *L. monocytogenes* per gram of raw fish from the unobservable prevalence of the lot *j* (*p_j_*). Such a procedure calls for two assumptions: (1) that *L. monocytogenes* cells are randomly distributed in fish (i.e., are not clustered), and (2) that the analytical weight was the same (25 g) in all detection assays carried out by the sources shown in Table 2. A further assumption if that a clean, degutted fish unit weighs 3800 g.

**Table 2:** *L. monocytogenes* prevalence in raw fish sampled at processing facilities.

Country	Product	Sample Size, *n*	Positive Enrichment, *s*	Prevalence (%)	Source *
Finland	Raw rainbow trout	35	0	0.00	Autio et al. [33]
Brazil	Raw salmon	255	105	41.2	Cruz et al. [34]
Ireland	Raw salmon	60	17	28.3	Dass [29]
Italy	Raw salmon	21	5	23.8	Di Ciccio et al. [35]
Finland	Raw fish	45	2	4.40	Markkula et al. [36]
	Fish before processing	212	9	4.20	
Poland	Incoming salmon	46	2	4.34	Medrala et al. [37]
	Incoming seatrout	26	4	15.4	
Finland	Raw fish	18	2	11.1	Miettinen et al. [38]
Norway	Salmon pre filleting	24	4	16.6	Rorvik et al. [39]
Denmark	Raw fish	12	0	0.00	Vogel et al. [40]
	Raw fish	18	0	0.00	

(*) Data extracted from the Pathogens-in-Foods Database [61], except for Cruz et al. [34].

A function, Lot2LotGen(), was built to generate a contamination matrix, *N*, of dimensions *r* × *c*, whose number of rows, *r*, represents the number of lots and can therefore be understood as number of iterations that will correspond to between-lot variability; the number of columns, *c*, represents the number of pre-filleting fish units of weight *Unit_size_* (3800 g) to be produced in the lot, considered as fixed and equal for all lots (cf. Appendix A.1).

#### 2.1.2. Storage of Pre-Fillet Fish

The primary processing module begins with storing gutted, clean fish prior to filleting. Even under very cold storage conditions*, L. monocytogenes* can still grow. Jia et al. [42] have provided models for this growth based on their studies, where they inoculated salmon with various strains of *L. monocytogenes* and stored them at temperatures ranging from 4 to 35 °C. They used a Lotka–Volterra-based equation to determine how temperature affects the growth rate and the initial delay before growth starts, considering the competitive effects of the fish’s natural microbiota.

The QRA model applies these findings to predict the growth rate and the initial delay period of the pathogen during storage, using a straightforward log-linear model to estimate the pathogen’s concentration at the end of the storage period. Jia et al. [42] also provided the maximum population density of *L. monocytogenes* in raw fish (9.20 log_10_ CFU/g). The model assumes a brief holding period for the fish before filleting, with storage temperature and duration modeled to vary according to Pert distributions. Specifically, temperature and storage time for each lot are sampled from Pert (−2, 0, 4) and Pert (0.5, 2.0, 6.0), respectively.

Additionally, an auxiliary function, sfGrowthLDP(), calculates the number of *L. monocytogenes* in a unit of raw fish after storage, based on constant temperature and time settings using validated models for maximum growth rate and delay duration [42,62]. This function aids in multiple storage assessments for raw fish, as detailed in Appendix A.2. The function sfRawFishStorage() then uses these calculations to stochastically simulate the growth of *L. monocytogenes* during cold storage, pulling data from a contamination matrix and the storage variability parameters listed in Appendix A.3.

#### 2.1.3. Filleting of Raw Fish

As early as 1995, Eklund et al. [47] and Rorvik et al. [39] observed that *L. monocytogenes* could be transferred from the exterior of fish to cut surfaces of fillets or sides, and concluded that filleting is a critical stage, since filleting tables, knives, and gloves of personnel could further spread the contamination. The representation of cross-contamination during fish slicing was regarded as relevant, because *L. monocytogenes* present on tables and cutting surfaces can adhere strongly after a short period of time, which entails the possibility that filleted fish become contaminated during the first stages of processing.

Aarnisalo et al. [44] carried out a study to investigate the transfer of *L. monocytogenes* from an inoculated slicing blade (slicer) to slices of gravad salmon, and from inoculated salmon fillet to the slicing machine and subsequently to slices of uninoculated fillets. A marked reduction in the counts from the slicing blade (5.9–9.0 log_10_ CFU/blade) to the fillets (1.6 log_10_ CFU/g) was observed after 39 slices; nonetheless, the first slices contained higher counts. Hoelzer et al. [43] utilized this bacterial transfer dataset, along with others produced from slicing meat and RTE meat products, to populate a compartment model consisting of four elements (slicer, chub, slice, and a bacterial loss bin). From this deterministic model, they produced various estimates of coefficients for slicing transfer (*a*) and for transfer from original contamination to the system (*e*) and derived thereof variability distributions regarding the transfer coefficients *a* and *e*. Additionally, Hoelzer et al. [43] proposed a probabilistic model of microbial transfer during a generic slicing process. This model and its parameters are employed in the present QRA to represent the transfer of *L. monocytogenes* cells during the filleting operation. A further assumption is that two fillets of weight 1300 g each can be obtained from one clean fish unit.

A function, sfSlicer(), was written to stochastically simulate the transfer of bacteria during this step. The function is fed by the outputs of the function sfRawFishStorage(), the weight of the fillet (*w_Fillet_*), the load of *L. monocytogenes* cells on the slicer (knife or blade) (*Init_Slicer_*), the parameters of the logistic distribution regarding the transfer coefficient *a* (*location_a_*, *scale_a_*), and the parameters of the normal distribution of the log_10_ coefficient *e* (*μ_loge_*, *σ_loge_*). The outputs of the function sfSlicer() are as follows: the contamination matrix of *L. monocytogenes* numbers in fish fillets (*N_Fillet_*), the previous expenditure of *L. monocytogenes* lag phase corresponding to those fillets (*WorkDone_s_*), the (unchanged) probability vector of contaminated lots after filleting *Prob_UnitPos_*, and the mean prevalence of contaminated lots after filleting (*P_Fillet_*) (cf. Appendix A.4).

#### 2.1.4. Holding-Off Time of Fish Fillets

The growth of *L. monocytogenes* in fish fillets during the holding-off stage is simulated. The objective of introducing a storage time after filleting is to allow for the transportation of fish fillets from the primary to the secondary processing facility, or, alternatively, for a short period of time before commencing processing (salting in the case of smoked fish or smearing with curing agents in the case of gravad fish). The latter case is represented in the present QRA model, assuming that the holding-off temperature (*Temp_hold_*, °C) and time (*time_hold_*, h) follow Pert distributions, *Pert (−2, 0, 4)* and *Pert (1, 2.0, 6.0)*, respectively. The maximum population density (MPD) of *L. monocytogenes* in raw fish fillets is assumed to be the same as in raw clean fish (9.20 log_10_ CFU/g).

The function sfRawFishStorage() and its auxiliary function sfGrowthLPD(), explained in detail in Appendix A.2 and Appendix A.3, are reused in the holding-off stage to stochastically estimate the growth of *L. monocytogenes* in raw fish fillets. In a lot, the fish fillets are considered to be exposed to the same *Temp_hold_* and *time_hold_*, which are sampled from the aforementioned Pert distributions. In addition to these parameters, the inputs of the function sfRawFishStorage() are the contamination matrix, *N_Fillet_*, the microbial work done after the first storage (*WorkDone_s_*, a matrix), the weight of the fillet (*w_Fillet_*, a scalar), and the maximum population density of *L. monocytogenes* in the fish fillet (*MPD*, a scalar), whereas the outputs are the contamination matrix after *L. monocytogenes* growth (*N_Hold_*) and the total microbial work done (*WorkDone_h_*, a matrix).

$$\left\{N_{Hold\;ij},\;{WorkDone}_{h\;ij}\right\}=sfGrowthLPD\left(N_{Fillet\;ij}\;,\;{time}_{hold\;i},\;{Temp}_{hold\;i},\;w_{Fillet},\;MPD,\;{WorkDone}_{s}\right)\;j=1,\;2\ldots ,{\;c}_{f}$$

sfRawFishStorage() returns unaffected the mean prevalence of contaminated lots of fish fillets (*P_Hold_*, a scalar) and the probability that the sampled lot is contaminated (*Prob_UnitPo_*_s_, a vector).

#### 2.1.5. Brining or Salting of Fish Fillets (Relevant to Smoked Fish)

In the processing of smoked fish, fish fillets can be salted in two ways: by dry-salting or by brining (i.e., injection of a saturated NaCl solution). Given that *L. monocytogenes* is able to survive in the salted fish due to its halotolerance, no microbial reduction or inactivation process is represented at this stage. Instead, opportunities for external or internal contamination during salting are contemplated. In the case of brining, recirculating brine is a source for contamination since *L. monocytogenes* can survive in NaCl solutions of up to 10% [63]. Gudmundsdottir et al. [46] and Gudbjornsdottir et al. [45] found *L. monocytogenes* at frequencies of 21.4% (3/14) and 8.7% (2/23), respectively, in injection brines, which supported the fact that brine containers and the brine itself may serve as reservoirs for *L. monocytogenes*. Based on these data, the QRA model assumed that, on a lot basis, the probability that the brine solution is contaminated (*Pcc_Brine_*) is 13.5% (5/37). When this event occurs, an internal contamination of the fish fillet is produced through the brine injected. Nonetheless, no data were found on the likely *L. monocytogenes* levels in contaminated brine solution, and therefore the numbers were assumed.

With regards to dry-salting, it is considered that cross-contamination can take place through tables or other surfaces during the smearing of fish fillets with salt/sugar/spices, at a certain probability, *Pcc_Smear_*. The probability *Pcc_Smear_* is assumed to be 3.9% [46]. Every fillet is subjected to the same *Pcc_Smear_* probability, and if the contamination event takes place, cells are partially transferred according to a transfer coefficient. The normal distribution parameters of the log_10_ transfer coefficient of *L. monocytogenes* (*TR_Smear_*) were taken from Hoelzer et al. [43] to represent the transfer from board to meat. Nonetheless, no data were found for the likely levels of *L. monocytogenes* on environmental elements in contact with fish fillets (*N_Surface_*) while dry-salting or smearing with other ingredients (gravlax curing).

Two auxiliary functions were built, sfBriningCC() and sfSmearingCC(), to model the cross-contamination during brining and dry-salting, respectively. The function sfBriningCC() stochastically simulates the potential internal contamination of fish fillets during brining by the injection of salt solution [64] (cf. Appendix A.5). The function sfSmearingCC() stochastically simulates the potential external contamination of fish fillets during dry salting when producing smoked fish, or during smearing with gravlax curing agents when producing gravad or any macerated fish (cf. Appendix A.6). Those two auxiliary functions could be used on their own, if we were to assume that all the lots of fish fillets were processed either through brine injection or through dry salting. The main function sfBrineORsaltCC() was conceived to allow for the inclusion of both types of salting in the QRA model. Thus, some of the lots of fish fillets will be subjected to brining (at the probability *p_Brine_*) and others to dry-salting (at the probability 1-*p_Brine_*) (cf. Appendix A.7).

#### 2.1.6. Smoking and Maturation of Salted Fish Fillets (Relevant to Smoked Fish)

The combination of hurdles that occur in the processing of smoked fish (salting, drying, and smoking) affect the homeostasis of *L. monocytogenes*; as they struggle to maintain their energy balance, they become metabolically exhausted during the subsequent step of maturation, causing the populations to drop [65]. Although the temperature during smoking is too low to eliminate *L. monocytogenes*, the phenolic compounds from smoke at a concentration of 20 ppm can inhibit *L. monocytogenes* [66].

In the literature, few studies investigating the overall effects of the individual processing steps for the production of smoked salmon have been undertaken for both types of salting (brine injection or dry-salting). Table 3 compiles the available information on the combined effect of cold-smoking and maturation on *L. monocytogenes*.

In each of those challenge studies, the conditions of smoking and the treatment prior to smoking were different. In the work of Neunlist et al. [49], smoking dry-salted salmon reduced *L. monocytogenes* counts by 0.5 log_10_ CFU/g; however, during subsequent maturation, the effect of salt and smoke continued, and the mean number of *L. monocytogenes* was further reduced by another 0.5 log_10_ CFU/g. Thus, the combination of the two steps significantly reduced the concentration of *L. monocytogenes* by 1.0 log_10_ CFU/g.

In the paper by Porsby et al. [48], the effect of smoking and maturation was carried out in salmon fillets that were salted either by brine injection or by dry-salting and in fresh unsalted salmon fillets as the control. From different challenge studies, they determined that smoking and maturation could reduce *L. monocytogenes* in up to 1.9 log_10_ after 24 h in brined fish, although they could not find statistical differences with salmon fillets that were dry-salted. Eklund et al. [47] inoculated *L. monocytogenes* in two different manners: on the surface, to simulate external contamination, and internally, to simulate contamination through brine injection; cold-smoking was the carried out at two distinct ranges of temperature: a lower temperature (17–21 °C) and a higher temperature (22–30 °C). The results of Elkund et al. [47] suggested that smoking and maturation may be more effective in reducing *L. monocytogenes* populations in dry-salted salmon fillets than in brine injected ones. In the experiment where cold-smoking was conducted at the higher temperatures of 22–30 °C on salmon samples that were inoculated (internally) via brine injection, smoking and maturation did not appear to have any effect on *L. monocytogenes*. These results agreed with Niedziela et al. [67], who earlier pointed out that dry-salting is more effective in controlling *L. monocytogenes* than brine injection. Dry-salting pulls water out of the flesh due to osmotic pressure. On the contrary, a brine-injected fillet will keep the water distributed in the flesh, and the surface water on the fish may even be pulled towards the higher salt concentration within the fish. As the surface is the part of the fish that tends to have higher concentrations of *L. monocytogenes*, the difference of losing surface water and keeping surface water will cause a different microbial concentration change in the fish.

Thus, the present QRA model estimates the log_10_ reduction factors due to smoking and maturation for brine-injected fish fillets and dry-salted fish fillets by calculating the mean and sample standard deviations of the values compiled in Table 3. The mean (*μ_rBrine_*) and standard deviation (*σ_RBrine_*) of the normally distributed log_10_ reduction factor for brined fish fillets (*R_Brine_*) are 0.871 and 0.807 log_10_, respectively, while the mean (*μ_rDrysalt_*) and standard deviation (*σ_RDrysalt_*) of the normally distributed log_10_ reduction factor for dry-salted fish fillets (*R_Drysalt_*) are 1.093 and 0.532 log_10_, respectively.

A function, sfSmoking(), was written to simulate the microbial process of inactivation of *L. monocytogenes* in the salted fish fillets subject to smoking and 18–24 h maturation (cf. Appendix A.8). Its inputs are the outputs of the function sfBrineORsaltCC() and the normal distribution parameters *μ_RBrine_*, *σ_RBrine_*, *μ_RDrysalt_*, and *σ_RDrysalt_* that describe the microbial log_10_ reduction in *L. monocytogenes* for brined or dry-salted fish fillets. These parameters are pre-defined, as described above.

#### 2.1.7. Smearing of Fish Fillets with Ingredients (Relevant to Gravad Fish)

Different procedures have been reported for the elaboration of gravad fish. According to Wiernasz [68], for gravlax production in France, salmon fillets are cured with a mix of salt, sugar, pepper, and dill during a period of 14 h at 6 °C, whereas Peiris et al. [63] explain that, in Sweden, salmon or rainbow trout are rubbed with a mixture of sugar, salt, and pepper and are covered with dill and stored in plastic bags at low temperature for 48 h. A longer procedure has been described by Cruz et al. [34]; salmon fillets are hand-rubbed with a commercial mixture of NaCl, sodium nitrate, and sodium nitrite and stored at 4 °C for 24 h in high-density polypropylene boxes. After the excess of salt is washed out with chlorinated water and the fillets are drained, a mixture of sugar, NaCl, ground white pepper, and dried dill is hand-rubbed into the flesh side of the fillets, and they are then stored for 24 h at 4 °C in the boxes. Fillets are then layered on stainless steel supports, sprayed with sweet wine, and ripened for 48 h at 4 °C.

In a processing line for gravlax, Cruz et al. [34] found that, amongst the food contact surfaces, the pathogen was present in 40% of the samples from salting boxes, ripening trolleys after 48 h in the cold room, and weighting trays, whereas a lower frequency of positive samples was found in the salting table (30%). Therefore, as for dry-salting in the production of smoked fish, during smearing with salt/sugar/spices in the production of gravad fish it is assumed that bacterial transfer can occur from tables or other surfaces, at a probability *Pcc_Smear_* (3.9% obtained from Gudmundsdottir et al. [46]). As with the smearing of fish with salt (Section 2.1.5), for the smearing of fish fillets with gravlax curing agents the same parameter values were employed: $\mu_{TRsmear}=-0.29$ and $\sigma_{TRSmear}=0.31$ for the normal distribution truncated on ]∞, 0] of the log_10_ transfer coefficient of *L. monocytogenes* (*TR_Smear_*) [43], and the load of *L. monocytogenes* on environmental elements in contact with a fish fillet (*N_Surface_* = 10 CFU).

The function sfSmearingCC() is used to stochastically simulate the potential external contamination of fish fillets during smearing with salt, sugar, and condiments. The function is described in detail in Appendix A.6. In the context of the processing of gravad fish, sfSmearingCC() is fed by the outputs of the holding-off storage function (*N_Hold_*, *Prob_UnitPo_*_s_, and *P_Hold_*) and *Pcc_Smear_* (0.039), $N_{surface}$ (100 CFU), μ_TRSmear_ (−0.29), and σ_TRSmear_, (0.31). The function provides as outputs the contamination matrix *N_Smear_*, the vector *Prob_UnitPos Smear_*, and the scalar *P_Smear_*.

#### 2.1.8. Maceration or Curing of Fish Fillets (Relevant to Gravad Fish)

Lopes et al. [50] performed a challenge test on traditional gravlax salmon to determine the effect of processing on *L. monocytogenes* counts. Salmon fillets, contaminated at a level of 5.5 log_10_ CFU/g, were premoistened with lemon juice and covered with a mixture of salt, brown sugar, black pepper, and dill and then cured at 4.5 °C. After 72 h of curing, the population of *L. monocytogenes* was reduced by 0.9 log_10_ CFU/g. In the work of Neunlist et al. [49], the salting step had a weaker effect on *L. monocytogenes* culturability of 0.5 log_10_ CFU/g. From this information, a log_10_ reduction factor for gravlax maceration of fish fillets (*R_Gravad_*) was derived, assuming that it followed a normal distribution with mean *μ_RGravad_* = 0.70 and standard deviation *σ_RGravad_* = 0.283 log_10_ CFU/g.

A function, sfMaceration(), was built to stochastically represent the reduction of *L. monocytogenes* concentration in fish fillets smeared with gravlax curing agents during cold maceration or curing. The inputs of this function are the contamination matrix *N_Smear_* and the normal distribution parameters *μ_RGravad_* and *σ_RGravad_*, defining the microbial log_10_ reduction in *L. monocytogenes* in macerating fish fillets smeared with curing ingredients.

#### 2.1.9. Slicing of Processed Fish Fillets

In the literature, a few environmental microbiological surveys have evidenced that slicing machines in RTE seafood processing facilities can be a source of *L. monocytogenes* spread. For instance, Di Ciccio et al. [35] repeatedly isolated *L. monocytogenes* serotypes 1/2a and 1/2b from slicer belts, distribution trays, slicing machines, and slicing covers for three years in a smoked-salmon production facility: out of 95 environmental samples tested, slicing machines (37%) and working tables (43%) had the highest frequencies of detection. In the USA, in a processing plant of catfish fillets, Chen et al. [69] determined that in 15% (7/45) of the sampling times, skinning, slicing, or blending equipment were contaminated with *L. monocytogenes*. In Ireland, Dass [29] isolated MLVA types *L. monocytogenes* serotypes c and b in the slicing machines over a one-year survey (2 positives out of 36). Thus, it was deemed necessary to represent cross-contamination during the slicing of processed fish fillets in the present QRA model. The function sfSlicer(), developed from the compartmental model of Hoelzer et al. [43] and used to simulate cross-contamination during fish filleting, is also employed at the slicing stage. In this case, the unit to be sliced is the processed fish fillet of weight *w_Fillet_* and the sliced unit is the smoked fish slice or the gravad fish slice, of weight *w_Slice_*.

The function sfSlicer(), already used during the filleting process and described in Appendix A.4, takes the outputs of the function sfSmoking() in the case of the QRA for smoked fish, or the outputs of the function sfMaturation() in the case of the QRA for gravad fish. In any case, the weight of a fish fillet (*w_Fillet_*) is 1300 g and the weight of a slice (*w_Slice_*) is 32.5 g. The parameters of the logistic distribution about the transfer coefficient *a* (*location_a_* = 0.07; *scale_a_* = 0.03), and the parameters of the normal distribution of the log_10_ coefficient *e* (*μ_loge_* = −2.12; *σ_loge_* = 0.85) remain the same as those used in the stage of filleting [43], whereas the load of *L. monocytogenes* cells on the slicer (*Init_Slicer_*) is set to 0.

#### 2.1.10. Packaging of Processed Fish Slices

There is no assumption of cross-contamination during the packaging of processes fish slices. A function, sfPackaging(), was written. It consists of a simple grouping of consecutive slices to produce a pack of *Slices_pack_* slices. It adds up the microbial load *N_Slice_*, corresponding to the group of slices, and the weight of the slices (pack). *Slices_pack_* is assumed to be 8 slices per pack; thus, the grouping produces a pack of weight *Unit_SizePack_*, which equals *Slices_pack_* × *w_Slice_* (8 × 32.5 = 260 g). The inputs of the function sfPackaging() are *N_Slice_* and *Slices_pack_*, and the output is the contamination matrix *N_Pack_*. The number of packs of end-product (*c_p_*) produced in a lot will then be *c_s_/Slices_pack_*. Thus, the output *N_Pack_* is a matrix of dimensions *r* lots by *c_p_* packs. The mean prevalence of contaminated packs, *P_Pack_*, returns the value of *P_Smoked_* for smoked fish packs, or the value of *P_Smear_* for gravad fish packs.

#### 2.1.11. Within-Lot Testing

The QRA model enables the testing of *L. monocytogenes* in food unit samples taken from a lot, according to a two-class or a three-class mixed sampling plan. In the two-class plan, *n* samples are randomly extracted and analyzed per lot. For each sample, a sub-sample of *g* grams is used in the enrichment essay, and the lot is rejected if more than *c* samples are positive in detection. In a three-class mixed sampling plan, samples are also enumerated, and the lot is rejected if more than *c* units are positive in detection or if at least one unit is found to have a concentration greater than a predefined concentration *M* [70]. It is assumed that the enumeration assay is conducted only on positive samples in detection, by direct plating of $g_{TestedEnum}$ g taken from the same sample.

A function, sfTesting(), was built to consider this selection step. Contaminated lots detected after testing are not removed from the matrix, and so the input matrix output *N_Pack_* is returned unchanged. The function fvTesting() only updates the probability for each lot to be put on the market, using the Bayes’ theorem (cf. Appendix A.10).

#### 2.1.12. Cold Chain

The characteristics of smoked fish and gravad fish—namely, intrinsic factors (pH, aw, preserving compounds, lactic acid protective cultures) and extrinsic factors (temperature, atmosphere in the package) do not preclude *L. monocytogenes* from growing [26]. If the pathogen is present on processed fish fillets, salting will not be sufficient to inhibit its growth. The slow development of *L. monocytogenes* relies upon cold temperatures and vacuum packaging, which reduces the pathogen’s growth due to anaerobic conditions and the inhibitory effect of competitive LAB. The cold chain step in the present QRA models encompasses the transportation from end processing to retail, the time at retail, and the transportation from retail to home.

In the cold chain segment of this study, the growth of *L. monocytogenes* and lactic acid bacteria (LAB), which can inhibit the pathogen, is modeled under various conditions. This module encompasses several key steps: Predictive models are utilized to determine the growth rates of both the pathogen and LAB in seafood, taking into account internal factors like pH and external factors such as temperature (refer to Appendix A.11 and Appendix A.12 for model details). A comprehensive growth model that includes initial growth delays is employed to estimate the concentrations of the pathogen and LAB at the conclusion of the cold chain (see Appendix A.13). The characteristics of the seafood that influence microbial growth rates are evaluated through a specific function (see Appendix A.14). The principal cold chain function integrates this data to simulate the growth of both microbes as the seafood progresses from production to retail (refer to Appendix A.15). Table 4 provides a list of the parameters used in these models, detailing their significance and data sources.

Briefly (see Appendix A for details), the function sfMejlholmDalgaard() deterministically computes the growth rate of *L. monocytogenes* in RTE seafood at given intrinsic and extrinsic characteristics, according to the secondary model based on the Gamma concept for lightly preserved seafood developed and validated by Mejlholm and Dalgaard [51,52,53,54,55,56] using the. Similarly, the function sfMejlholmDalgaardLAB() deterministically estimates the growth rate of LAB in RTE seafood at given environmental characteristics, according to the secondary model developed in [54]. The function sfGrowthJameson() estimates the numbers of *L. monocytogenes* and LAB (CFU) in RTE seafood at given environmental characteristics after a certain time period. The function is based on the Baranyi–Roberts-based Jameson-effect competition model put forward by Giménez and Dalgaard [57], supplemented with the Gamma (*γ*) interaction parameter later proposed by Moller et al. [58]. By default, *γ* is set to 1. Eventually, assuming Jameson-effect microbial competition and allowing for the presence of a lag phase, the function sfColdChain() stochastically estimates the concentration of *L. monocytogenes* and LAB populations in the RTE seafood product at the end of the cold chain distribution (i.e., arrival at consumer’s home).

#### 2.1.13. Home Storage

During home storage, *L. monocytogenes* and LAB continue to grow. Endrikat et al. [76] represented the home storage time in days by a Weibull distribution with shape parameter 1.14 and scale parameter 18.39. This information was translated into a Pert distribution for the minimum value *Time_Home min_* (0.73 h as the 2.5% percentile of the Weibull distribution) and the mode *Time_Home mode_* (70 h as the mode of the Weibull distribution). The maximum home storage time, *Time_Home max_*, consists of a best guess of 35 days (840 h). Likewise, a Pert distribution regarding the home storage temperature (*Temp_Home_*) was approximated from the normal distribution used in Pouillot et al. [20], truncating at the 2.5% and 97.5% percentiles to obtain the minimum (*Temp_Home min_* = 1.12) and the maximum (*Temp_Home max_* = 12.9) parameters, respectively. The mean of the normal distribution was set as the mode (*Temp_Home mode_* = 7.0). In addition, a low correlation between home storage time and temperature was quantified as rank correlation by Pouillot et al. [19], which will be used in the present QRA model (*CorTimeTemp_Home_* = −0.12).

The simultaneous growth of *L. monocytogenes* and LAB are simulated using the function ColdChain() and its auxiliary functions (cf. previous section), as performed for the distribution module of cold chain, including interactions [77]. Values of home storage time (*time_Home ij_*) and temperature (*Temp_Home ij_*) are sampled for every RTE seafood pack (i.e., different consumers), targeting the rank correlation value of *CorTimeTemp_Home_* [78].

$${time}_{Home\;ij}\;\sim \;Pert\;\left({time}_{Home\;min},{time}_{Home\;mode},{time}_{Home\;max}\right)\;i=1,\;2,\;\ldots ,\;r;j=1,\;2,\;\ldots ,\;c_{p}\;{Temp}_{Home\;ij}\;\sim \;Pert\;\left({Temp}_{Home\;min},{Temp}_{Home\;mode},{Temp}_{Home\;max}\right)\;i=1,\;2,\;\ldots ,\;r;j=1,\;2,\;\ldots ,\;c_{p}$$

The growth rates *of L. monocytogenes* (*μ_LM ij_*) and LAB (*μ_LAB ij_*) in RTE seafood for every pack *ij* stored at the temperature *Temp_Home ij_* are then computed by the functions sfMejlholmDalgaard() and sfMejlholmDalgaardLAB(), respectively, using the product’s environmental characteristics already generated in the cold chain stage by the function sfCharacteristics().

Next, the function sfGrowthJameson() is executed to produce the numbers of *L. monocytogenes* and LAB in the RTE seafood units at the point of consumption (*N_Home LM_*, *N_Home LAB_* in CFU).

$$\left\{N_{Home\;LM\;ij},\;N_{Home\;LAB\;ij},\;{\ln q}_{Home\;LM\;ij},{\ln q}_{Home\;LAB\;ij}\;\right\}=sfGrowthJameson\;\left(N_{cc\;LM\;ij}\;,\;{N_{cc\;LAB\;ij},\;time}_{Home\;ij},\;{exp(q}_{cc\;LM\;ij}),\;{exp(q}_{cc\;LAB\;ij}),\;\mu_{LM\;ij},\;\mu_{LAB\;ij},\;{MPD}_{LM\;i},{MPD}_{LAB\;i},\gamma ,{Unit}_{SizePack}\;\right)\;i=1,\;2,\;\ldots ,\;r;\;j=1,\;2\ldots ,{\;c}_{p}$$

#### 2.1.14. Portioning Before Consumption

During serving at home, it is assumed that the consumer removes a serving size *Serv_size_* (g) from the pack of RTE seafood of net weight *Unit_SizePack_* g. Then, the number of *L. monocytogenes* cells in this small unit can be considered to be a sample from a Beta-binomial distribution. Furthermore, a moderate clustering of *L. monocytogenes* cells in the RTE seafood contained in the pack is assumed (dispersion, *b* = 1; [59]). The dispersion parameter and the number of portions that can be obtained from a pack are assumed to be independent of the microbial numbers. A function, sfPortioning(), was developed to simulate the microbial process of partitioning (cf. Appendix A.16).

### 2.2. Hazard and Risk Characterisation

Several dose-response relationships based on the exponential model are available for *L. monocytogenes* [16,21,26,79,80]. The dose-response model chosen to estimate the risk of listeriosis per serving of RTE seafood is that of Pouillot et al. [79] for the elderly population (>65 years old) with unknown underlying conditions. According to this model, the probability *r* that an ingested *L. monocytogenes* cell causes an invasive listeriosis follows a log _10_ normal distribution, with mean −12.83 and standard deviation 1.62. The function DRQuick() from the doseresponsemodels R package [79] was employed to estimate the marginal probabilities of invasive listeriosis in the elderly population, *RiskServing_ij_*, from the input matrix of doses, *N_Portion ij_*. The mean risk for every lot *i* (*RiskLot_i_*) was calculated as a risk averaged across servings, *j*, and weighed by the lot-specific probability, *Prob_UnitPos Tested i_*:

$${RiskLot}_{i}=\frac{\sum_{j=1}^{c_{p}}{RiskServing}_{ij}\times {Prob}_{UnitPos\;Tested\;i}}{c_{p}}$$

### 2.3. QRA Model’s Ouputs

The model’s outputs were summarized at three stages: end of processing, point of consumption, and risk characterization. The descriptors at the *end of processing* were computed from the prevalence and the contamination matrix outputs of the function sfTesting() and included the following: (1) descriptive statistics (mean, median, and 95% confidence interval) of the mean concentration of *L. monocytogenes* (CFU/g) in the fraction of contaminated lots; (2) the prevalence of contaminated packs; and (3) the probability that a contaminated pack contains more than 10 CFU *L. monocytogenes* per g RTE seafood.

The model’s descriptors at the *point of consumption* were estimated from the outputs of the function sfColdChain(), applied to depict home storage, and encompassed the following: (1) descriptive statistics (mean, median, and 95% confidence interval) of the concentration of *L. monocytogenes* in any serving; (2) the prevalence of contaminated servings; (3) the probability that a contaminated serving contains more than 10 CFU *L. monocytogenes* per g RTE seafood; and (4) the probability that a contaminated serving contains more than 100 CFU *L. monocytogenes* per g RTE seafood.

The descriptors for risk characterization include the summary statistics, mean, median, and 2.5, 97.5, and 99.5 percentiles of the lot-level mean risk per serving *RiskLot*.

### 2.4. QRA Model’s Functionality: Reference and What-If Scenarios

To illustrate the utility of the QRA model, reference scenarios were separately run for the three types of RTE seafood studied: smoked brine-injected fish, smoked dry-salted fish, and gravad fish. In addition, five what-if scenarios were run for each type of RTE seafood, which assessed the following: (1) reduction of the between-lot mean prevalence of *L. monocytogenes*; (2) absence of initial contamination load on filleting knives; (3) no transfer of contamination during salting (smoked fish) or smearing with ingredients (gravad fish); (4) application of protective LAB cultures; and (5) reduction of storage temperature at home.

(a) Reference, constituted by the baseline scenarios of the three RTE seafood products, with parameter values supported as much as possible by current data and, in their absence, by reasonable assumptions.
(b) Reduction of *L. monocytogenes* prevalence in a lot of incoming fish, assessed by setting the parameter $\hat{\alpha }$ of the Beta distribution regarding prevalence to half its original value (0.437).
(c) Reduction in cross-contamination during filleting, assessed by assuming that filleting knives are cleaned/disinfected after filleting every fish, and therefore setting the parameter *Init_Slicer_* of the function sfSlicer() to zero.
(d) Absence of contamination during salting or smearing of fish fillets, represented by a zero probability of contamination during brine injection (*Pcc_Brine_* = 0%) for smoked brined fish and zero probability of contamination during smearing with salt or curing agents (*Pcc_Smear_* = 0%) for both smoked dry-salted fish and gravad fish.
(e) Application of protective cultures when processing fish fillets, represented by an increase in the mean lot concentration of LAB in RTE seafood after packaging (${\bar{C}}_{0\;LAB}$) by 5 log_10_ CFU/g. Therefore, the minimum (${\bar{C}}_{0\;LAB\;min}$), mode (${\bar{C}}_{0\;LAB\;mode}$), and maximum (${\bar{C}}_{0\;LAB\;max}$) parameters defining the Pert distribution regarding ${\bar{C}}_{0\;LAB}$ were set to 4.00, 5.28, and 6.60 log_10_ CFU/g, respectively, for the RTE products.
(f) Lower home storage temperature, represented by a decrease of 1.5 °C in the mode and maximum storage temperature at home (Temp_Home mode_ = 5.5 and Temp_Home max_ = 11.4).

The reference and model scenarios for the three RTE seafood products were run by setting an initial contamination matrix size of r = 5000 lots and c =100 fish units in the function Lot2LotGen().

### 2.5. QRA Model’s Implementation

All the functions described in Section 2.1 were programmed in R (R Core Team [81]) and compiled in the package qraLm, which can be installed from the Github repository: https://github.com/WorldHealthOrganization/qraLm, accessed on 10 September 2024. The reference manual can be found at: https://WorldHealthOrganization.github.io/qraLm/reference/, accessed on 10 September 2024.

## 3. Results and Discussion

### 3.1. Reference Scenario and Comparison with Other QRA Models

The outcomes of the references scenarios suggested that, despite the high prevalence of lots contaminated with *L. monocytogenes* at the end of processing—38.7% for smoked brined fish, 34.4% for smoked dry-salted fish, and 52.2% for gravad fish—the mean concentrations in the fraction of contaminated lots would be very low (0.0028 CFU/g for smoked brined fish, 0.0021 CFU/g for smoked dry-salted fish, and 0.0029 CFU/g for gravad fish; Table 5). The prevalences of contaminated packs leaving the processing facilities were estimated at 8.14%, 6.49%, and 11.14%, respectively, although virtually no pack would contain numbers higher than 10 CFU/g (Table 5). Higher levels of contamination in cold-smoked and salt-cured salmon produced in Finland were predicted by a Markov-chain-based model developed by Pasonen et al. [30]. They estimated that ~10% of the contaminated packs exceeded the 100 CFU/g limit, whereas the prevalence of contaminated packs was 22% (95% CI: 20–25%). Although the assumptions of such a QRA differed in many instances from our model, it is plausible that their higher exposure estimates primarily obey the greater *L. monocytogenes* initial prevalence levels from the Finnish survey data used as inputs in their QRA model (in the range of 15.7–31.8% in the period between 2004 and 2010). By contrast, in our QRA model the mean of the between-lot *L. monocytogenes* prevalence was 14.85%, as modelled by a Beta (0.8741, 5.880) distribution, built upon survey data from multiple countries. Nonetheless, despite these differences, our model did coincide with that of Pasonen et al. [30] in the prediction that *L. monocytogenes* contaminated packs tend to have low bacterial concentrations, but on a few rare occasions the concentrations at the point of consumption would be in the order of hundreds of CFU/g (Table 6).

According to the present QRA model, at the end of processing a production lot of gravad fish would be more likely to be contaminated with *L. monocytogenes* than a production lot of smoked fish, and, in turn, if the smoked fish was salted by brine-injection the probability of lot contamination would be higher than if dry-salted. The prevalence of *L. monocytogenes* predicted by our QRA model for smoked (6.40–8.14%) and gravad fish packs (11.15%) are fairly in concordance with the joint results of EFSA’s EU-wide baseline survey and monitoring data, which encountered overall prevalence values of 8.42% (219/2602) in cold-smoked fish and 11.11% (30/270) in gravad fish, as compiled by Pérez-Rodríguez et al. [24].

Outcomes from other epidemiological surveys are also comparable to our simulation findings for gravad fish; namely, the incidence of 15% for cold stored gravlax from a Brazilian salmon processing plant (Cruz et al. [34]) and the incidence of 8% in gravlax fish from Icelandic markets [82]. In terms of enumeration estimates at the point of consumption (Table 6), the simulations predicted mean concentrations of *L. monocytogenes* in any serving (contaminated or not) of 103.6 CFU/g for smoked brined fish and 125.9 CFU/g for smoked dry-salted fish, and a higher mean concentration, at 162.6 CFU/g, for gravad fish, although it is worth mentioning that these high mean estimates are a result of a few highly contaminated servings, as implied by their 95% confidence intervals ([0–0.2261 CFU/g], [0–0.1197 CFU/g], and [0–3.3787 CFU/g], respectively). In the same order of magnitude of these intervals fell the median values of *L. monocytogenes* concentration found for cold-smoked fish (2.48 CFU/g) and for gravad fish (3.34 CFU/g) predicted by Pérez-Rodríguez et al. [24].

In their short-scope QRA model, the consumption of gravad fish was linked to greater exposure than smoked fish, in agreement with our model. Nonetheless, our model’s estimate for the level of exposure from a contaminated serving of smoked fish (log_10_ (103.6/0.0459) = 3.35 log_10_ CFU/g, worked out from Table 5) was considerably higher than the estimates from the QRA models of *L. monocytogenes* in contaminated servings of French cold-smoked salmon (mean: 1.38 log_10_ CFU/g; Pouillot et al. [19]) and smoked salmon consumed in Navarra (mean: 2.25 log_10_ CFU/g; Garrido et al. [27]).

On the other hand, our mean concentrations in any serving of smoked fish (103.6 CFU/g and 125.9 CFU/g) are close to the output of a QRA model of *L. monocytogenes* in Irish cold-smoked salmon, which predicted a mean concentration in any serving of 71.6 CFU/g (as determined by the product of the mean prevalence of contaminated servings of 18% and the mean concentration in contaminated servings of 10^2.6^ CFU/g) (Dass [29]).

The probability that a serving is contaminated with *L. monocytogenes* followed a decreasing order: 7.35% for gravad fish, 4.59% for smoked brined fish, and 3.52% for smoked dry-salted fish (Table 6). The same rank order was observed for the probabilities that a contaminated serving contains more than 10 CFU/g and 100 CFU/g. These were in the ranges 1.00–2.60% and 0.32–1.04%, respectively. At least three-fold higher probabilities of *L. monocytogenes* in RTE fish servings were found by the EFSA’s generic short-scope QRA model [16], which estimated that proportions of 8.01% cold-smoked fish servings and 4.66% gravad fish servings would exceed a concentration of 100 CFU/g. These considerable deviations may stem from the following assumptions: (1) initial prevalences of *L. monocytogenes* (at end of processing) that were higher (17.4% for cold-smoked fish and 12.2% for gravad fish) than the throughputs of our model (6.49–8.14% for smoked fish and 11.14% for gravad fish); (2) initial concentrations of *L. monocytogenes* in the contaminated fraction (at end of processing) that were higher (0.867 log_10_ CFU/g for cold-smoked fish and 1.011 log_10_ CFU/g for gravad fish) than the throughputs of our model (0.0021–0.0028 CFU/g for smoked fish and 0.0029 CFU/g for gravad fish); and (3) serving sizes (49–66 g for smoked fish and 129–154 g for gravad fish in the elderly population) that were higher than the serving size of one slice (32.5 g) assumed in our model.

**Figure 2:**
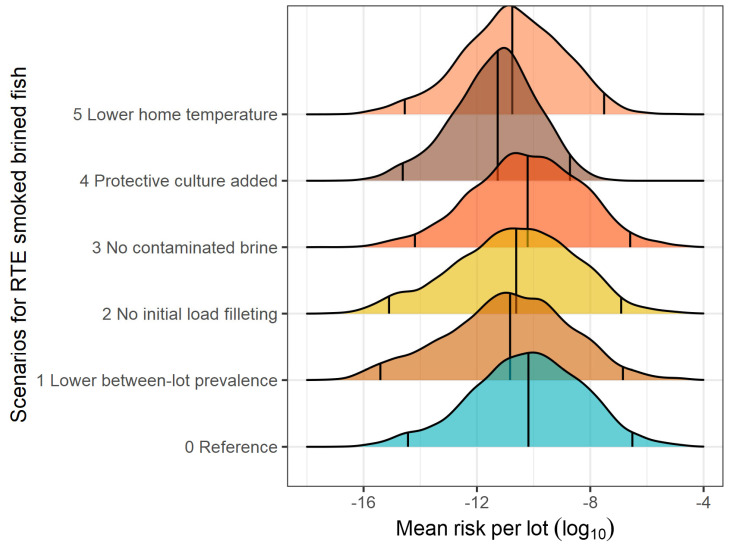
Lot-level mean risk (log_10_) associated with the consumption of a 32.5-g serving (slice) of RTE smoked brine-injected fish, as evaluated for the reference and selected scenarios. Vertical lines on density plots indicate the median and interquartile range limits.

**Figure 3:**
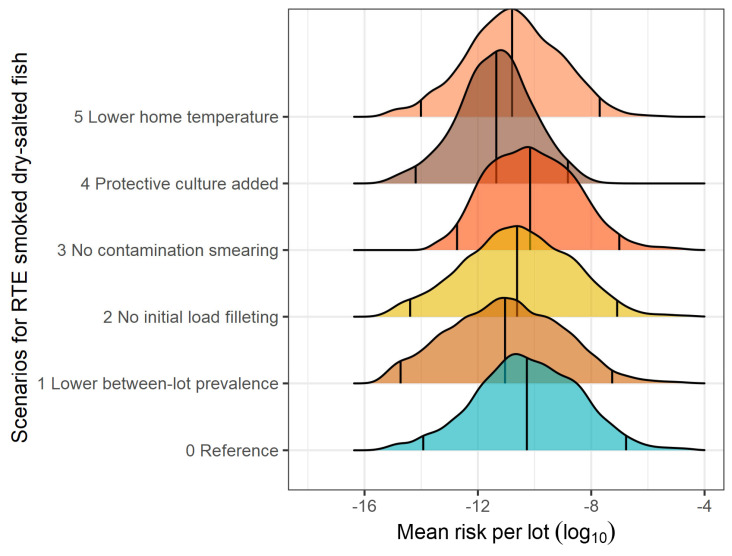
Lot-level mean risk (log_10_) associated with the consumption of a 32.5-g serving (slice) of RTE smoked dry-salted fish, as evaluated for the reference and selected scenarios. Vertical lines on density plots indicate the median and interquartile range limits.

**Figure 4:**
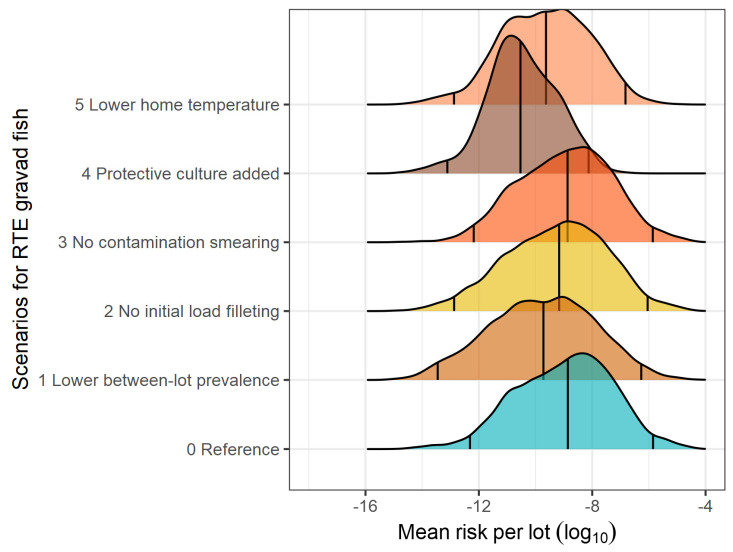
Lot-level mean risk (log_10_) associated with the consumption of a 32.5-g serving (slice) of RTE gravad fish, as evaluated for the reference and selected scenarios. Vertical lines on density plots indicate the median and interquartile range limits.

The present QRA model revealed a high between-lot variability in the risk of listeriosis associated with the consumption of a slice of RTE fish product (Table 7). The heterogeneity in the mean risk from lot to lot is represented in Figure 2, Figure 3 and Figure 4 for smoked brine-injected fish, smoked dry-salted fish, and gravad fish, respectively. The mean lot risks of listeriosis per serving were estimated at 9.751 × 10^−8^ (median 6.572 × 10^−11^) for smoked brined fish, 9.634 × 10^−8^ (median 5.352 × 10^−11^) for smoked dry-salted fish, and 2.086 × 10^−7^ (1.376 × 10^−9^) for gravad fish (Table 7). On a serving basis of one slice, the mean risk linked to gravad fish was 0.33 or 0.34 log_10_ higher than those of smoked brined or dry-salted fish. Similarly, the EFSA’s generic QRA model [16] calculated a 1.12 log_10_ higher risk of listeriosis for sliced reduced-oxygen packaged gravad fish in comparison to sliced reduced-oxygen packaged cold-smoked fish in the elderly population, using the same dose-response model (Pouillot et al. [80]). Nevertheless, the median risk values were considerably higher in the EFSA model (4.37 × 10^−9^ for cold-smoked fish and 5.86 × 10^−8^ for gravad fish), which is likely to have arisen from the higher values of the model inputs discussed above.

In comparison to our QRA simulation, other models attained very different mean risk estimates for cold-smoked salmon, namely, the model of Pouillot et al. [20] (1.3 × 10^−6^ in the French elderly population) and the model of Garrido et al. [27] (4.17 × 10^−10^ in the normal and immunocompromised population of Navarra, Spain). Dass [29], in his listeriosis QRA model from Irish cold-smoked salmon, estimated a median risk in the high-risk population (6.165 × 10^−11^), which was in closer agreement with our median risk estimates for smoked fish.

For comparison with the EFSA’s model outputs of number of annual illnesses [16], the mean risk estimates of our model were used to compute the mean number of invasive listeriosis in the 28 EU MS per year, based on the same estimated number of annual servings in the elderly population (1.99 × 10^10^ servings of smoked fish and 8.35 × 10^9^ servings of gravad fish). According to these values, our QRA model would predict a mean of 1940 annual cases of listeriosis linked to the consumption of smoked fish and 1741 annual cases linked to gravad fish. Using the same consumption data, EFSA [16] estimated a considerably lower number of listeriosis cases, at 201 and 230 cases in the elderly EU population linked to cold-smoked fish and gravad fish, respectively.

It is worth highlighting that the listeriosis dose-response model chosen has a high impact on the final risk estimate, and hence on the number of cases predicted. For instance, if the dose-response model of FAO-WHO [26] for increased susceptible population had been used, the present QRA model would have produced lower mean risk per serving estimates of 3.566 × 10^−9^ (median 4.511 × 10^−13^) for the smoked brined fish and 5.599 × 10^−9^ (median 9.776 × 10^−12^) for the gravad fish. These would have in turn predicted 72 and 48 annual listeriosis cases in the EU, 28 linked to these two products, respectively, assuming a consumption of 2.03 × 10^10^ servings of smoked fish and 8.53 × 10^9^ servings of gravad fish in the increased susceptible population (elderly plus pregnant population, taken from Pérez-Rodríguez et al. [24]).

### 3.2. What-If Scenarios

Among the what-if scenarios evaluated, reducing the prevalence of *L. monocytogenes* in fish entering the processing facilities would produce a decrease in the proportion of contaminated lots at the end of processing in about 40–53% for the three RTE seafood products, followed by the use of disinfected filleting knives (13–15%; Table 5). The absence of bacterial transfer during brine injection or smearing with ingredients would generate a lower effect on the prevalence of contaminated lots (decreasing it in 2.5–3.5% in smoked brined fish and gravad fish) because the frequency of contamination during salting assumed in the reference scenario is already low. Likewise, the what-if scenario that would reduce the prevalence of contaminated packs at the end of processing the most would be the reception of incoming fish with a lower prevalence of *L. monocytogenes*; this would reduce the proportion of outgoing contaminated packs from 8.14% to 4.97% in smoked brined fish, from 6.49% to 3.11% in smoked dry-salted fish, and from 11.14% to 5.42% in gravad fish (Table 5). The effectiveness of the what-if scenarios in decreasing the *L. monocytogenes* concentration in contaminated lots is, however, less evident in the mean and median estimates, given the high variability in *L. monocytogenes* contamination from lot to lot; however, the lowering effect can be appreciated in the 97.5th percentile. A scenario of absence of contamination during brine injection or smearing with ingredients would not practically lower the mean concentration of *L. monocytogenes* in contaminated lots at the end of processing, as it would lower the prevalence of contaminated lots and contaminated packs. As deduced by the 97.5th percentile in the concentration of contaminated lots (0.2261 CFU/g in smoked brined fish, 0.1197 CFU/g in smoked dry-salted fish, and 3.3787 CFU/g in gravad fish in the reference scenario), halving the prevalence of incoming fish into processing lines would be far more effective (to 0.0605 CFU/g, 0.0206 CFU/g, and 0.4191 CFU/g, respectively) than keeping filleting knives free of *L. monocytogenes* at all times (to 0.1195 CFU/g, 0.0642 CFU/g, and 1.8050 CFU/g, respectively; Table 5).

The effectiveness of the use of protective LAB cultures and the lower storage temperature at home can be compared with the other scenarios through the exposure assessment simulation outputs at the point of consumption (Table 6). Whereas reducing the contamination prevalence of incoming fish was the scenario that produced the highest reduction (52–54%) in the prevalence of contaminated servings (from 4.59% to 2.67% in smoked brined fish, from 3.52% to 1.65% in smoked dry-salted fish, and from 7.35% to 3.53% in gravad fish), in terms of *L. monocytogenes* numbers, the greatest control was achieved by the use of protective cultures, which led to the lowest *L. monocytogenes* concentration in any serving, and, as a consequence, the lowest probabilities of finding counts greater than 10 CFU/g or 100 CFU/g in a contaminated serving. However, the efficacy of the use of LAB cultures was found to depend on the RTE seafood product: for the smoked brined/dry-salted fish, the application of protective cultures produced a 2000/3000-fold decrease in the mean *L. monocytogenes* concentration in any serving and an 83/85% decrease in the probability that a contaminated serving contains more than 10 CFU/g (Table 6). In gravad fish, the efficacy of using protective culture was lower, causing a 780-fold decrease in the mean *L. monocytogenes* concentration in any serving and a 78% decrease in the probability that a contaminated serving contains more than 10 CFU/g. Nonetheless, considering the 100 CFU/g limit, there was no real difference in LAB culture effectiveness between the types of RTE product, since in all products the application of protective cultures led to a ~96% reduction in the probability of finding *L. monocytogenes* counts greater than 100 CFU/g in a contaminated serving.

After the use of protective cultures, the next “most effective scenario” to control *L. monocytogenes* up to the point of consumption was the maintenance of the RTE seafood product to lower storage temperatures at home. Reductions in the mean concentration in any serving and its 97.5th percentile were in the order of 13-to-32-fold and 2.1-to-3.3-fold, respectively. From *L. monocytogenes* mean concentrations at the point of consumption of 103.6 CFU/g (97.5th pct 0.2261 CFU/g), 125.9 CFU/g (97.5th pct 0.1197 CFU/g), and 162.6 CFU/g (97.5th pct 3.378 CFU/g) in the smoked brined fish, smoked dry-salted fish, and gravad fish, respectively, the sole proper storage practice taken by the consumers would reduce the concentrations to 6.4923 CFU/g (97.5th pct 0.0994 CFU/g), 3.8733 CFU/g (97.5th pct 0.0567 CFU/g), and 12.486 CFU/g (97.5th pct 1.0267 CFU/g) in the smoked brined fish, smoked dry-salted fish, and gravad fish, respectively. The frequency of contaminated servings above the 100 CFU *L. monocytogenes* per gram of serving would also be reduced by 50-60% in the three RTE products. In terms of prevalence of contaminated servings, however, the level of reduction attained by keeping colder home temperatures would be nearly the same as that of applying protective cultures (4.13% versus 4.16% for smoked brined fish, 3.18% versus 3.20% for smoked dry-salted fish, and 6.75% versus 6.60% for gravad fish; Table 6).

An inverse result on the relative importance of these two scenarios (colder home temperatures and added protective LAB cultures) in the concentration of *L. monocytogenes* in the servings of smoked salmon was purported by the listeriosis QRA model of Pouillot et al. [19], where they found that the mean storage temperature at the consumer phase (*p* = 10^−20^) was a stronger determinant of exposure than the initial background microbiota (LAB) concentration (*p* = 0.002). Other QRA models whose outcomes helped to reinforce the relevance of improving consumer’s awareness of the correct storage conditions of RTE seafood and avoid temperature abuse were the ones developed by Dass [29], Garrido et al. [27], Pérez-Rodríguez et al. [24], and Pasonen et al. [30].

In our QRA model, the two scenarios relative to decreasing cross-contamination during secondary processing—namely, no initial contamination load on filleting knives and no bacterial transfer during brine injection or smearing with ingredients—were effective but, among the five what-if scenarios evaluated, yielded the least extent of reduction in *L. monocytogenes* prevalence and concentrations in RTE seafood servings (Table 6). In both smoked brined fish and gravad fish, maintaining filleting knives free of *L. monocytogenes* was more effective than the absence of contamination during brining/smearing in decreasing the mean concentration in any serving (23–25% reduction against 3.4–3.7%), the 97.5th percentile in the mean concentration in any serving (47% reduction against 0.5–1.2%), the proportion of contaminated servings with more than 10 CFU/g *L. monocytogenes* (9.0% reduction versus 1.6–1.9%), and the proportion of contaminated servings with more than 100 CFU/g *L. monocytogenes* (7.1–9.6% reduction versus 2.5–2.8%). The opposite outcome was encountered for the smoked dry-salted fish, where avoiding bacterial transfer during dry-salting would be more effective than keeping filleting knives disinfected, producing a reduction by 41% in the mean concentration in any serving versus 17%, a reduction by 44% in the prevalence of contaminated servings versus 22%, and a reduction by 40% in the proportion of servings with *L. monocytogenes* concentrations greater than 10 or 100 CFU/g against 8.0% or 9.3%, respectively.

In the three RTE seafood products, it was evident—within the constraints of the model assumptions—that the cross-contamination that could occur during processing (filleting and salting or smearing with curing agents) would be a less important determinant of the consumer’s exposure to *L. monocytogenes* when compared to the maintenance of proper cold storage at home or to the pathogen’s occurrence in fish entering processing.

The impact of the what-if scenarios on the between-lot mean risk per serving of RTE seafood product can be visualized in the density curves of Figure 2, Figure 3 and Figure 4, which also show the shifts in the 2.5th and the 97.5th percentiles. Among the scenarios evaluated, the application of protective LAB cultures (which increases the initial LAB counts by 5 log_10_ CFU/g) would be the most effective strategy to hinder the growth of *L. monocytogenes*, thereby reaching a mean risk reduction of 2.55 log_10_ for smoked brined fish, 2.76 log_10_ for smoked dry-salted fish, and 2.31 log_10_ for gravad fish. This strategy also reduced, to the greatest extent, the median (by >1.0 log_10_) and the highest (97.5 and 99.5th by >2.2 log_10_) percentiles of the mean lot risk posed by the three RTE products (Table 7). Decreasing the home storage temperature by 1.5 °C (mode and maximum) turned out to be the second most important risk control strategy, causing a risk reduction level in the range of 0.52–0.76 log_10_ for the 50th percentile, 0.93–1.00 log_10_ for the 97.5th percentile, and 0.98–1.25 log_10_ for the 99.5th percentile among the three RTE products. In terms of mean risk reduction, keeping the RTE products at lower temperatures would bring about a mean risk reduction of 0.94 log_10_ in smoked brined fish, 1.14 log_10_ in smoked dry-salted fish, and 0.88 log_10_ in gravad fish. A very similar prediction was computed in a QRA model in Irish cold-smoked salmon (Dass [29]), where, by fixing the storage temperature from 3–10 °C to 4 °C, the mean risk was decreased by 1.10 log_10_ in the increased susceptibility population. In comparison to our findings, lower mean risk reductions have been reported by QRA models in analogous scenarios, such as decreasing the distribution temperature profiles by 1–2 °C caused a risk reduction of 0.60 log_10_ (Pérez-Rodríguez et al. [24]), decreasing home storage temperature from 7 °C to 4 °C caused a median risk reduction of 0.52 log_10_ (Pasonen et al. [30]), and all domestic temperatures with a mean temperature of 5.4 °C led to a risk reduction of 0.46 log_10_ (Garrido et al. [27]).

In smoked dry-salted fish and gravad fish, decreasing the mean lot prevalence by half was the what-if scenario that followed in effectiveness, as attested by the mean risk reductions of 0.28 and 0.27 log_10_, respectively. The risk lot medians linked to these two RTE products were also reduced by 0.77 and 0.86 log_10_, respectively, whereas the high percentiles were reduced by 0.40- 0.50 log_10_. This would imply that, if contamination was assumed to be carried over between lots (within the same processing facility), the mean lot risk would in turn be increased.

Nonetheless, in smoked brine-injected fish, the risk reduction attained by decreasing the prevalence of contaminated lots was low (0.05 log_10_) and comparable to the effect of avoiding contamination during brine injection (0.02 log_10_ risk reduction), although the former provided a slightly higher risk reduction, as also attested by the median (1.478 × 10^−11^ versus 6.164 × 10^−11^), the 97.5th percentile (1.422 × 10^−7^ versus 2.566 × 10^−7^), and the 99.5th percentile (2.881 × 10^−6^ versus 3.287 × 10^−6^). The relatively lower importance of the scenario of reduced initial prevalence, found in the present QRA, is in agreement with the findings of other authors, who determined that the strategies that reduced growth (such as the use of protective cultures or the reduced storage temperature tested in our simulations) present a greater impact on the final risk than strategies that reduce the occurrence [19,22,83]. Furthermore, measures aimed at reducing the initial prevalence of *L. monocytogenes* in fish are not well documented.

The strategy of keeping filleting knives free of *L. monocytogenes* at all times (i.e., the very ideal scenario of having no initial load on knives before filleting every fish) rendered a mean risk reduction that was comparable across the three RTE products evaluated—0.15 log_10_ in smoked brined fish, 0.11 log_10_ in smoked dry-salted fish, and 0.10 log_10_ in gravad fish—with fairly important reductions in the median risk (0.30–0.43 log_10_) and high percentiles (0.10–0.40 log_10_). In relation to the absence of bacterial transfer during fillet smearing with salt or with curing agents, the effectiveness of this scenario to diminish the final risk of listeriosis depended on the type of product, which in gravad fish provided a very negligible mean risk reduction of 0.01 log_10_, while in dry-salted fish the mean risk reduction was higher at 0.20 log_10_ (very close to the effect of lowering the prevalence of contaminated lots). This difference is explained by the fact that, although the parameters assumed for smearing were the same in both products, gravad fish has intrinsic characteristics that are more favorable for *L. monocytogenes* growth. Thus, in gravad fish, the growth of *L. monocytogenes* after the product leaves the factory has a contribution to final risk that nearly overcomes the risk reduction effect of the absence of contamination during fillet smearing.

Taking together the results of the five scenarios across the three RTE seafood products, it became clear that the consumer’s conventional measure of maintaining the product at the recommended cold temperatures is very effective in decreasing the risk of listeriosis (overall mean risk reduction of ~1.00 log_10_). Nevertheless, since this measure is at the same time the hardest to implement (and impossible to control), strategies that are placed at the processing level must be considered, namely reducing the prevalence of *L. monocytogenes* in incoming un-filleted fish (overall mean risk reduction of ~0.20 log_10_) and/or slowing down the pathogen’s growth in the product. The latter can be achieved by the use of protective cultures (overall mean risk reduction of ~2.56 log_10_) or even the modification of the physicochemical properties of the product (lower water activity, addition of organic acids or other growth inhibitors [84]). Avoiding cross-contamination is an effective control measure; however, the model has evidenced that, once *L. monocytogenes* has entered the processing lot, minimizing the chances for cross-contamination during filleting, salting, or smearing with curing agents would result in a comparatively small risk reduction, which is lower for gravad fish (0.01–0.10 log_10_) compared to smoked fish (0.02–0.15 log_10_).

## 4. Conclusions and Perspectives

We have developed a robust four-module quantitative risk assessment (QRA) model of *L. monocytogenes* in ready-to-eat (RTE) fish products. This model, composed of 18 functions and a total of 128 parameters, is freely available and designed with flexibility in mind. It provides, for the first time, the ability to check for the efficacy of batch testing on risk. Our findings highlight the critical importance of strict hygiene practices during processing, the benefits of using protective LAB cultures, and the necessity of proper storage conditions by consumers to effectively mitigate the risk of listeriosis. The model’s inherent adaptability ensures that food safety authorities can tailor interventions to meet specific needs, enhancing food safety measures for various RTE seafood products.

As new data become available, the parameters can be updated, and the functions, programmed in R software, can be reassembled to represent different seafood products. For example, to adapt the model for exposure assessment of sashimi, the smoking function (sfSmoking()) can be omitted, while for ceviche, it would be appropriate to remove the smoking function and add the maceration function (sfMaceration()), adjusting the parameters to suit these specific raw fish dishes.

This QRA model is intended to assist food safety authorities worldwide in obtaining risk estimates and evaluating risk management options tailored to their specific RTE seafood chains and product characteristics. Moreover, the model facilitates the evaluation of a variety of intervention strategies (such as the use of bacteriocinogenic cultures, organic acids, extended smoking periods) by allowing adjustments to interaction parameters in the Jameson model or modifying growth rates for *L. monocytogenes* and lactic acid bacteria (LAB). This QRA model not only provides a framework for assessing the risk of listeriosis from the consumption of smoked and gravad fish but also offers the potential to apply its methodology to a broader range of seafood products, thereby supporting global efforts in improving food safety.

## Figures and Tables

**Table 1:** Sequence of stages, microbial processes represented, data sources, assumptions, and corresponding functions coded in R for the construction of the exposure assessment model of *Listeria monocytogenes* (LM) in RTE smoked and gravad fish.

Module	Stage	Microbial Process	Assumptions	Sources	Function in R
Primary processing	Generation of contaminated lots of pre-fillet (pre-processed) fish	None	LM prevalence in fish units is modelled from data found in incoming fish sampled at primary processing. Since LM numbers in incoming fish were generally low (<10 CFU/g), LM concentration in fish was calculated from the expected proportion of non-zeroes under a Poisson distribution.	Autio et al. [33], Cruz et al. [34], Dass [29], Di Ciccio et al. [35], Markkula et al. [36], Medrala et al. [37], Miettinen et al. [38], Rorvik et al. [39], Vogel et al. [40], Jarvis [41]	Lot2LotGen()
	Storage	Growth	LM in pre-filleted fish is assumed to follow the kinetics of a cocktail of CICC 21632 (serotype 1/2a), CICC 21633 (serotype 1/2a), CICC 21635 (serotype 4b), and CICC 21639 (serotype 1/2a) LM inoculated in raw salmon flesh. Lag phase is considered.	Jia et al. [42]	sfRawFishStorage(), served by sfGrowthLDP()
	Filleting	Cross-contamination	LM is transferred to the fillets during the slicing process of the raw whole fish using a compartmental model defined by two variability distributions: “a”, the transfer rate between slicer blade (or filleting knife) and product, and “e”, the transfer rate from the original contamination to the slicing system. Distribution parameters were obtained from published data.	Hoelzer et al. [43], Aarnisalo et al. [44]	sfSlicer()
	Holding-off time	Growth	The same predictive microbiology model as in Storage, yet the LM growth and the exhaustion of the lag phase were followed up in this stage.	Jia et al. [42]	sfRawFishStorage(), served by sfGrowthLDP()
Secondary processing	Brining or salting (smoked fish)	Cross-contamination	Fish fillets can be salted either through brine injection or by dry salting. Internal contamination by brining may occur during the injection of saline solution due to brine containers or the brine itself serving as reservoirs for LM, at a given probability. For dry-salted fillets, it is assumed that external contamination can occur during the procedure of smearing salt on fish fillets if surfaces are contaminated with LM, at a given probability. A published distribution for LM transfer rate is assumed.	Gudbjornsdottir et al. [45], Gudmundsdottir et al. [46] Hoelzer et al. [43]	sfBrineORsaltCC(), served by sfBriningCC() and sfSmearingCC()
	Smoking and maturation (smoked fish)	Inactivation	Salting, drying, and smoking produce a slight reduction in LM, which is different if fish fillets were brine-injected or dry-salted. The LM log_10_ reduction in brine-injected fish fillets was assumed to be that of the combined results of inoculation experiments in smoked salmon, first salted through brine injection, and then submitted to cycles of cold-smoking between 6 and 8 h until a total maturation time of 18–24 h. The LM log_10_ reduction in dry-salted fish was assumed to be that of the combined results of inoculation experiments on the surface of dry-salted salmon, determined before and after a total maturation time between 18 and 24 h.	Eklund et al. [47], Porsby et al. [48] Eklund et al. [47], Porsby et al. [48], Neunlist et al. [49]	sfSmoking()
	Smearing (gravad fish)	Cross-contamination	During smearing of fish fillets with salt, sugar, and spices, external contamination can occur if surfaces are contaminated with LM, at a given probability. A published distribution for LM transfer rate is assumed.	Hoelzer et al. [43]	sfSmearingCC()
	Maceration (gravad fish)	Inactivation	The maceration of fish smeared with gravlax curing agents is assumed to reduce the populations of LM, according to the results of a study where inoculated raw salmon was smeared with salt, brown sugar, black pepper, and dill and left to macerate at 4.5 °C for 72 h.	Lopes et al. [50]	sfMaceration()
	Slicing	Cross-contamination	The same compartmental model as in Filleting, but used to produce slices from smoked or gravad fish fillets.	Hoelzer et al. [43], Aarnisalo et al. [44]	sfSlicer()
	Packaging	Mixing	No cross-contamination is assumed during packaging. Consecutive slices of recently-sliced RTE smoked or gravad fish are gathered into packages of end-product.	-	sfPackaging()
	Within-lot testing	None	At a given probability, any lot of RTE smoked or gravad fish can be subjected to sampling and testing, according to a two-class or three-class microbiological sampling plan.	-	sfTesting()
Distribution	Cold chain	Growth	LM in smoked or gravad fish, as affected by populations of lactic acid bacteria (LAB), are assumed to grow during the various cold chain logistics stages, including transportation to retail, display at retail, and transportation to home. Specific growth rates for LM and LAB are estimated from secondary models, using validated kinetic parameters. LM numbers follow an extended Jameson-effect competition model, which uses an interaction Gamma parameter.	Mejlholm and Dalgaard [51,52,53,54,55], Mejlholm et al. [56] Gimenez et al. [57], Moller et al. [58]	sfColdChain(), served by sfMejlholmDalgaard(), sfMejlholmDalgaardLAB(), sfGrowthJameson()
Consumer’s handling	Home storage	Growth	The same as in Cold chain, following up the growth.	Mejlholm and Dalgaard [51,52,53,54,55], Mejlholm et al. [56], Gimenez et al. [57], Moller et al. [58]	sfColdChain(), served by sfMejlholmDalgaard(), sfMejlholmDalgaardLAB(), sfGrowthJameson()
	Portioning	Partitioning	The consumer is assumed to take a number of RTE fish slices from the pack. LM cells present in a contaminated pack are assumed to be moderately clustered within the package.	Nauta [59]	sfPortioning()

**Table 3:** Reduction in *L. monocytogenes* concentration in salted salmon fillets due to smoking and maturation, taken from challenge tests.

Type of Salting	Source	Conditions of Smoking and Maturation	Before Treatment (log_10_ CFU/g)	After Treatment (log_10_ CFU/g)	Reduction (log_10_ CFU/g)
Brine injection	Porsby et al. [48]	Cold smoking, 24 °C in cycles of 6 h	3.00 ± 0.3	2.50 ± 1.1	0.50
			3.30 ± 0.4	1.80 ± 0.9	1.50
			2.90 ± 0.2	1.00 ± 0.0	1.90
			3.30 ± 0.1	1.80 ± 0.6	1.50
			3.00 ± 0.1	1.10 ± 0.3	1.90
	Elkund et al. [47]	Cold smoking, 17–21 °C × 18 h	2.36	1.41	0.95
			3.34	2.63	0.71
		Cold smoking, 22–30 °C × 18 h	2.11	2.34	−0.23
			3.28	3.38	−0.10
			4.57	4.49	0.08
Dry-salting	Porsby et al. [48]	Cold smoking, 24 °C in cycles of 6 h	3.30 ± 0.2	1.30 ± 0.7	1.80
			3.10 ± 0.2	1.40 ± 0.7	1.70
	Neunlist et al. [49])	Liquid smoking and maturing, 4 °C × 24 h	5.70	4.70	1.00
	Elkund et al. [47]	Cold smoking, 17–21 °C × 18 h	2.04	0.83	1.21
			2.56	1.11	1.45
		Cold smoking, 22–30 °C ×18 h	0.11	−0.39	0.50
			1.04	0.54	0.50
			2.42	1.84	0.58

**Table 4:** Parameters used for modelling the simultaneous growth of *L. monocytogenes* and lactic acid bacteria (LAB) in RTE seafood during cold chain distribution and home storage.

Type of Parameter	Parameter	Definition (Unit)	Value or Distribution	Source
Relative to kinetic parameters of LM in RTE seafood	$\mu_{LM\;ref}$	Optimum growth rate of LM (h^−1^)	0.419	Mejlholm and Dalgaard [51]
	*MPD*_LM_	Maximum population density of LM (log_10_ CFU/g)	*MPD*_LM min_ = 6.60 *MPD*_LM mode_ = 7.36 *MPD*_LM max_ = 8.20 *MPD*_LM_ ~ Pert (*MPD*_LM min,_ *MPD*_LM mode,_ *MPD*_LM max_)	Pérez-Rodríguez et al. [24]
	*h*_0 LM_	Parameter regarding the initial physiological state of LM (-)	*μ*_h0_ = 2.8 *σ*_h0_ = 4.6 *h*_0 LM_ ~ Normal (μ_h0_, σ_h0_), *h*_0_ > 0	Couvert et al. [71]: q_0_ then obtained by (1/(exp(*h*_0_)−1))
Relative to kinetic parameters of LAB in RTE seafood	$\mu_{LAB\;ref}$	Optimum growth rate of LAB (h^−1^)	0.583	Mejlholm and Dalgaard [54]
	MPD_LAB_	Maximum population density of LAB (log_10_ CFU/g)	*MPD*_LAB min_ = 8.0 *MPD*_LAB mode_ = 8.5 *MPD*_LAB max_ = 9.0 *MPD*_LAB_ ~ Pert (*MPD*_LAB min,_ *MPD*_LAB mode,_ *MPD*_LABM max_)	MPD_LAB min_ from Mejlholm and Dalgaard [54]
	*q*_0 LAB_	Parameter regarding the initial physiological state of LAB (-)	ln *q*_0 LABmin_ = −12 ln *q*_0 LABmode_ = 2.73 ln *q*_0 LABmax_ = 1.26 *q*_0 LAB_~exp{ Pert (ln *q*_0 LABmin_, ln *q*_0 LABmode_, ln *q*_0 LABmax_)}	Couvert et al. [71]
Relative to smoked fish characteristics	${\bar{C}}_{0\;LAB\;SF}$	Between-lot mean concentration of LAB in smoked fish after packaging (log_10_ CFU/g)	${\bar{\bar{C}}}_{0\;LAB\min SF}$ = −1.00 ${\bar{C}}_{0\;LAB\mathrm{mode}SF}\;$ = 0.28 ${\bar{C}}_{0\;LAB\max SF\;}$ = 1.60 ${\bar{C}}_{0\;LAB\;SF}$ ~ Pert (${\bar{\bar{\bar{C}}}}_{0\;LAB\min SF}$, ${\bar{\bar{C}}}_{0\;LAB\mathrm{mode}SF},\;{\bar{C}}_{0\;LAB\max SF\;})$	Wiernasz et al. [72]
	*pH*_SF_	pH of smoked fish (-)	*pH*_minSF_ = 5.8 *pH*_modeSF_ = 6.1 *pH*_maxSF_ = 6.5 *pH*_SF_~Pert (*pH*_minSF,_ *pH*_modeSF,_ *pH*_maxSF_)	*pH*_minSF_ from Mejlholm and Dalgaard [51] *pH*_modeSF_ from Porsby et al. [48] *pH*_maxSF_ from Hwang and Sheen [73]
	*NaCl*_SF_	NaCl content in smoked fish (% wb)	*NaCl*_minSF_ = 1.5 *NaCl*_modeSF_ = 3.4 *NaCl*_maxSF_ = 5.3 *NaCl*_SF_~Pert (*NaCl*_minSF,_ *NaCl*_modeSF,_ *NaCl*_maxSF_)	*NaCl*_minSF_ from FAO-WHO [26] *NaCl*_modeSF_ from Porsby et al. [48], Mejlholm and Dalgaard [51], FAO-WHO [26], and Orozco [74] *NaCl*_maxSF_ from Mejlholm and Dalgaard [51]
	*Phe*_SF_	Phenol compound in smoked fish (ppm)	*Phe*_minSF_ = 5 *Phe*_modeSF_ = 10 *Phe*_maxSF_ = 22 *Phe*_SF_~Pert (Phe_minSF_, *Phe*_modeSF_, Phe_maxSF_)	*Phe*_minSF_ from Leblanc et al. [72] *Phe*_modeSF_ from Hwang and Sheen [73], Porsby et al. [48], Mejlholm and Dalgaard [51], FAO/WHO [26], Leblanc et al. [75], and Eklund et al. [47] *Phe*_maxSF_ from Porsby et al. [48]
	*CO*2 _equi SF_	CO_2_ concentration at equilibrium in the packaging of smoked fish (fraction)	*CO*2_equi SF min_ = 0.25 *CO*2_equi SF mode_ = 0.25 *CO*2_equi SF max_ = 0.30 *CO*2_equi SF_~Pert (*CO*2_equi SF min_, *CO*2_equi SF mode_, *CO*2_equi SF max_)	Mejlholm and Dalgaard [51]
	Others: *Nit*_SF_, *LA*_tot GF_, *AA*_tot SF_, *BA*_tot SF_, *CA*_tot SF_, *DA*_tot SF_, *SA*_tot SF_	Nitrites concentration (ppm) and lactic acid, acetic acid, benzoic acid, citric acid, diacetate, lactic acid, and sorbic acid concentrations in water phase (ppm)	Allow for minimum, mode, and maximum for each compound to be sampled from Pert distribution. Values of zero set to all.	
Relative to gravad fish characteristics	${\bar{C}}_{0\;LAB\;GF}$	Between-lot mean concentration of LAB in gravad fish after packaging (log_10_ CFU/g)	${\bar{\bar{C}}}_{0\;LAB\min GF}$ = −1.00 ${\bar{C}}_{0\;LAB\mathrm{mode}GF}\;$= 0.28 ${\bar{C}}_{0\;LAB\max GF\;}$= 1.60 ${\bar{C}}_{0\;LAB\;GF}$ ~ Pert (${\bar{\bar{\bar{C}}}}_{0\;LAB\min GF}$, ${\bar{\bar{C}}}_{0\;LAB\mathrm{mode}GF},\;{\bar{C}}_{0\;LAB\max GF\;})$	Wiernacz et al. [72]
	*pH*_GF_	pH of gravad fish (-)	*pH*_minGF_ = 6.1 *pH*_modeGF_ = 6.2 *pH*_maxGF_ = 6.3 *pH*_SF_~Pert (*pH*_minSF,_ *pH*_modeSF,_ *pH*_maxSF_)	Mejlholm and Dalgaard [51] and Orozco [74]
	*NaCl*_GF_	NaCl content in gravad fish (% wb)	*NaCl*_minGF_ = 3.0 *NaCl*_modeGF_ = 3.2 *NaCl*_maxGF_ = 3.4 *NaCl*_GF_~Pert (*NaCl*_minGF,_ *NaCl*_modeGF,_ *NaCl*_maxGF_)	Mejlholm and Dalgaard [51] and Aarnisalo et al. [44]
	*Phe*_GF_	Phenol compound in gravad fish (ppm)	*Phe*_minSF_ = 0 *Phe*_modeSF_ = 0 *Phe*_maxSF_ = 5 *Phe*_GF_~Pert (Phe_minGF_, *Phe*_modeGF_, Phe_maxGF_)	Mejlholm and Dalgaard [51]
	*CO*2 _equi GF_	CO_2_ concentration at equilibrium in the packaging of gravad fish (fraction)	*CO*2_equi GF min_ = 0.25 *CO*2_equi GF mode_ = 0.25 *CO*2_equi GF max_ = 0.30 *CO*2_equi GF_~Pert (*CO*2_equi SF min_, *CO*2_equi SF mode_, *CO*2_equi SF max_)	Mejlholm and Dalgaard [51]
	Others: *Nit*_GF_, *LA*_tot GF_, *AA*_tot GF_, *BA*_tot GF_, *CA*_tot GF_, *DA*_tot GF_, *SA*_tot GF_	Nitrite concentration and lactic acid, acetic acid, benzoic acid, citric acid, diacetate, lactic acid, and sorbic acid concentrations in water phase (ppm)	Allow for minimum, mode and maximum for each compound to be sampled from Pert distribution. Values of zero set to all.	-
Relative to cold chain distribution	*time*_CC_	Time elapsed between end of production and arrival of the product at home (h)	*time*_CC min_ = 12 *time*_CC mode_ = 144 *time*_CC max_ = 720 *time*_CC_~Pert (*time*_CC min_, *time*_CC mode_, *time*_CC max_)	FDA-FSIS [23]
	*Temp*_CC_	Average temperature between end of production and arrival of the product at home (°C)	*Temp*_CC min_ = 0.28 *Temp*_CC mode_ = 4.60 *Temp*_CC max_ = 7.00 *Temp*_CC_~Pert (*Temp*_CC min_, *Temp*_CC mode_, *Temp*_CC max_)	From Normal (4.6, 2.2) °C in Pouillot et al. [20]
	*CorTimeTemp*_CC_	Correlation between time and temperature during cold chain	−0.16	Pouillot et al. [19]
Relative to home storage	*Time*_Home_	Storage time at home (h)	*Time*_Home min_ = 0.73 *Time*_Home mode_ = 70 *Time*_Home max_ = 840 for smoked fish and 528 for gravad fish *Time*_Home_ ~ Pert (*time*_Home min_, *time*_Home mode_, *time*_Home max_)	Minimum and mode values from Weibull (shape = 1.14, scale = 18.39) days in Endrikat et al. [76] Maximum is best guess: 35 days for smoked fish and 22 days for gravad fish
	*Temp*_Home_	Storage temperature at home (°C)	*Temp*_Home min_ = 1.12 *Temp*_Home mode_ = 7.0 *Temp*_Home max_ = 12.9 *Temp*_CC_~Pert (*Temp*_Home min_, *Temp*_Home mode_, *Temp*_Home max_)	From Normal (7, 3) °C in Pouillot et al. [20]
	*CorTimeTemp*_Home_	Correlation between time and temperature at home storage	−0.12	Pouillot et al. [19]

**Table 5:** Simulation outcomes of the exposure assessment model of *L. monocytogenes* in RTE seafood products at the end of processing for the reference and selected what-if scenarios. Simulations ran for 5000 lots consisting of 100 fish units each, where packs of final product weigh 260 g. Prevalence estimates are shown as proportions (scale 0–1).

Scenario	Mean Counts (CFU/g) in Contaminated Lots (Mean, Median, [95% CI])	Prevalence of Contaminated Lots	Prevalence of Contaminated Packs	P (*N* > 10 CFU/g in a Contaminated Pack) *
**Smoked brined fish**				
Reference	0.0028; 0.0017 [1.18 × 10^−4^–0.0130]	0.3870	0.0814	0
Lower prevalence of contaminated lots	0.0023; 0.0014 [9.49 × 10^−5^–0.0097]	0.2343	0.0497	0
No initial load on filleting knives	0.0023; 0.0012 [7.69 × 10^−5^–0.0117]	0.3298	0.0652	0
No contamination during brining	0.0028; 0.0017 [1.20 × 10^−4^–0.0129]	0.3767	0.0792	0
Addition of LAB culture	0.0028; 0.0017 [1.18 × 10^−4^–0.0130]	0.3870	0.0814	0
Lower home storage temperature	0.0028; 0.0017 [1.18 × 10^−4^–0.0130]	0.3870	0.0814	0
**Smoked dry-salted fish**				
Reference	0.0021; 0.0013 [1.28 × 10^−4^–0.0095]	0.3443	0.0649	0
Lower prevalence of contaminated lots	0.0016; 0.0009 [7.18 × 10^−5^–0.0072]	0.1611	0.0311	0
No initial load on filleting knives	0.0018; 0.0010 [8.71 × 10^−5^–0.0085]	0.2927	0.0512	0
No contamination during smearing	0.0012; 0.0005 [3.58 × 10^−5^–0.0063]	0.3554	0.0406	0
Addition of LAB culture	0.0021; 0.0013 [1.28 × 10^−4^–0.0095]	0.3443	0.0649	0
Lower home storage temperature	0.0021; 0.0013 [1.28 × 10^−4^–0.0095]	0.3443	0.0649	0
**Gravad fish**				
Reference	0.0029; 0.0020 [4.25 × 10^−4^–0.0102]	0.5215	0.1114	0
Lower prevalence of contaminated lots	0.0022; 0.0016 [2.66 × 10^−4^–0.0080]	0.3124	0.0542	0
No initial load on filleting knives	0.0023; 0.0016 [2.41 × 10^−4^–0.0091]	0.4521	0.0886	0
No contamination during smearing	0.0026/0.0018 [3.97 × 10^−4^–0.0100]	0.5031	0.1137	0
Addition of LAB culture	0.0029; 0.0020 [4.25 × 10^−4^–0.0102]	0.5215	0.1114	0
Lower home storage temperature	0.0029; 0.0020 [4.25 × 10^−4^–0.0102]	0.5215	0.1114	0

(*) In all scenarios, P (*N* > 100 CFU/g in a contaminated pack) = 0.

**Table 6:** Simulation outcomes of the exposure assessment model of *L. monocytogenes* in RTE seafood products at the point of consumption for the reference and selected what-if scenarios. Simulations ran for 5000 lots consisting of 100 fish units each, where packs of final product weigh 260 g and a serving is a slice of product (32.5 g). Prevalence estimates are shown as proportions (scale 0–1).

Scenario	Counts (CFU/g) in Any Serving (Mean, Median, [95% CI])	Prevalence of Contaminated Servings	P (N > 10 CFU/g in a Contaminated Serving)	P (*N* > 100 CFU/g in a Contaminated Serving)
**Smoked brined fish**				
Reference	103.6; 0.00 [0–0.2261]	0.0459	0.0122	0.0040
Lower prevalence of contaminated lots	103.4; 0.00 [0–0.0605]	0.0267	0.0110	0.0037
No initial load on filleting knives	76.39; 0.00 [0–0.1195]	0.0453	0.0111	0.0035
No contamination during brining	100.9; 0.00 [0–0.2139]	0.0444	0.0120	0.0039
Addition of LAB culture	0.0548; 0.00 [0–0.0886]	0.0416	0.0021	0.0002
Lower home storage temperature	6.4923; 0.00 [0–0.0994]	0.0413	0.0064	0.0016
**Smoked dry-salted fish**				
Reference	125.9; 0.00 [0–0.1197]	0.0352	0.0100	0.0032
Lower prevalence of contaminated lots	58.58; 0.00 [0–0.0206]	0.0165	0.0082	0.0027
No initial load on filleting knives	104.0; 0.00 [0–0.0642]	0.0274	0.0092	0.0029
No contamination during smearing	74.26; 0.00 [0–0.0763]	0.0210	0.0060	0.0019
Addition of LAB culture	0.0340; 0.00 [0–0.0508]	0.0320	0.0015	8.1 x 10^−5^
Lower home storage temperature	3.8733; 0.00 [0–0.0567]	0.0318	0.0052	0.0012
**Gravad fish**				
Reference	162.6; 0.00 [0–3.3787]	0.0735	0.0260	0.0104
Lower prevalence of contaminated lots	85.84; 0.00 [0–0.4191]	0.0353	0.0213	0.0085
No initial load on filleting knives	124.9; 0.00 [0–1.8050]	0.0579	0.0237	0.0094
No contamination during smearing	156.5; 0.00 [0–3.3617]	0.0744	0.0255	0.0101
Addition of LAB culture	0.2100; 0.00 [0–0.4503]	0.0660	0.0057	0.0005
Lower home storage temperature	12.486; 0.00 [0–1.0267]	0.0675	0.0157	0.0052

**Table 7:** Statistics of the mean risk of invasive listeriosis per lot of RTE seafood products in the elderly population for the reference and selected what-if scenarios. The logarithm base 10 of the mean risk reduction attained by each scenario in comparison to the reference one is shown (log_10_ RR).

Scenario	Mean	Median	2.5 pct	97.5 pct	99.5 pct	log_10_ RR
**Smoked brined fish**						
Reference	9.751 × 10^−8^	6.572 × 10^−11^	3.693 × 10^−15^	3.064 × 10^−7^	3.836 × 10^−6^	-
Lower prevalence of contaminated lots	8.778 × 10^−8^	1.478 × 10^−11^	3.911 × 10^−16^	1.422 × 10^−7^	2.881 × 10^−6^	0.05
No initial load on filleting knives	6.920 × 10^−8^	2.431 × 10^−11^	7.494 × 10^−16^	1.233 × 10^−7^	2.272 × 10^−6^	0.15
No contamination during brining	9.272 × 10^−8^	6.164 × 10^−11^	6.477 × 10^−15^	2.566 × 10^−7^	3.287 × 10^−6^	0.02
Addition of LAB culture	2.718 × 10^−10^	5.399 × 10^−12^	2.409 × 10^−15^	1.918 × 10^−9^	8.972 × 10^−9^	2.55
Lower home temperature	1.112 × 10^−8^	1.769 × 10^−11^	2.842× 10^−15^	3.082 × 10^−8^	2.113 × 10^−7^	0.94
**Smoked dry-salted fish**						
Reference	9.634 × 10^−8^	5.352 × 10^−11^	1.183 × 10^−14^	1.703 × 10^−7^	4.087 × 10^−6^	-
Lower prevalence of contaminated lots	5.113 × 10^−8^	9.071 × 10^−12^	1.868 × 10^−15^	5.483 × 10^−8^	1.513 × 10^−6^	0.28
No initial load on filleting knives	7.428 × 10^−8^	2.415 × 10^−11^	4.059 × 10^−15^	8.223 × 10^−8^	2.386 × 10^−6^	0.11
No contamination during smearing	5.984 × 10^−8^	6.953 × 10^−11^	1.835 × 10^−13^	9.772 × 10^−8^	2.608 × 10^−6^	0.20
Addition of LAB culture	1.693 × 10^−10^	4.462 × 10^−12^	6.363 × 10^−15^	1.502 × 10^−9^	5.888 × 10^−9^	2.76
Lower home temperature	6.899 × 10^−9^	1.607 × 10^−11^	9.780 × 10^−15^	1.991 × 10^−8^	1.817 × 10^−7^	1.14
**Gravad fish**						
Reference	2.086 × 10^−7^	1.376 × 10^−9^	4.863 × 10^−13^	1.402 × 10^−6^	9.436 × 10^−6^	-
Lower prevalence of contaminated lots	1.133 × 10^−7^	1.900 × 10^−10^	3.531 × 10^−14^	5.372 × 10^−7^	3.815 × 10^−6^	0.27
No initial load on filleting knives	1.623 × 10^−7^	6.810 × 10^−10^	1.344 × 10^−13^	9.013 × 10^−7^	7.841 × 10^−6^	0.10
No contamination during smearing	2.037 × 10^−7^	1.347 × 10^−9^	6.725 × 10^−13^	1.368 × 10^−6^	1.036 × 10^−5^	0.01
Addition of LAB culture	1.019 × 10^−9^	2.940 × 10^−11^	7.731 × 10^−14^	7.457 × 10^−9^	2.977 × 10^−8^	2.31
Lower home temperature	2.761 × 10^−8^	2.343 × 10^−10^	1.328 × 10^−13^	1.500 × 10^−7^	9.657 × 10^−7^	0.88

## Data Availability

The original contributions presented in the study are included in the article; further inquiries can be directed to the corresponding author.

## Appendix A

### Appendix A.1. The Function Lot2LotGen

For every lot *i* = {*1*, *2*, …, *r*}, a value of prevalence *P_i_* is sampled from the between-lot prevalence distribution $Beta\;(\hat{\alpha },\;\hat{\beta })$ $\left(\hat{\alpha }=0.8741,\;\hat{\beta }=5.880\right)$. The probability that lot *i* is contaminated (*Prob_UnitPos_*) is estimated as

$${Prob}_{UnitPos\;i}=1-P\left(s_{i}=0\right)$$

assuming that the number of contaminated fish units, *s_i_*, in a given lot, *i*, follows a Binomial distribution:

$$s_{i}\;\sim \;Binomial\left(c,P_{i}\right)$$

The mean prevalence of contaminated lots (*P*) is calculated as the complement of the Beta-binomial probability evaluated at *s* = 0,

$$P=1-\frac{\Gamma \left(c+\beta \right)}{\Gamma \left(c+\alpha +\beta \right)}$$

where $\Gamma \left(x\right)$ is the Gamma function. To produce the contaminated fish units, the mean numbers of *L. monocytogenes* per gram of fish (*λ_i_*, CFU/g) is calculated for every lot *i* from the Poisson assumption,

$$\lambda_{i}=-\frac{ln\left(1-P_{i}\right)}{w}$$

where *w* is the analytical sample weight used for detection analysis, assumed to be 25 g. The total number of *L. monocytogenes* in a contaminated lot (*N_tot i_*) is then sampled from a Poisson distribution,

$$N_{tot\;i}\;\sim \;Poisson\left(\lambda_{i}\times {Unit}_{size}\times c\right),\;{\;N}_{tot\;i}>0$$

Such total bacterial cells are then randomly distributed among the fish units of a lot, according to a multinomial distribution, assuming that all fish units have the same probability $\begin{array}{ccc} p_{1}=p_{2}= & \ldots & =p_{c} \end{array}=1/c$ of being contaminated,

$${\left[\begin{array}{ccc} N_{1}\;N_{2} & \ldots & N_{c} \end{array}\right]}_{i}\;\sim \;Multinomial\left(N_{tot\;i}\;,\;{\left[\begin{array}{ccc} p_{1}\;p_{2} & \ldots & p_{c} \end{array}\right]}_{i}\right)\;i=1,\;2,\;\ldots ,\;r$$

Thus, the resulting matrix *N* contains the numbers of *L. monocytogenes* cells in *c* units of fish (horizontal dimension) in a sample of *r* contaminated lots (i.e., where $\sum_{i}N_{i}>0$) (vertical dimension). The outputs of the function Lot2LotGen() are as follows: the contamination matrix (*N*), the mean prevalence of contaminated lots (*P*, a scalar), and the probability that the sampled lot is contaminated (*Prob_UnitPo_*_s_, a vector).

### Appendix A.2. The Function sfGrowthLDP

First, for a given temperature *Temp* and *time*, the maximum growth rate (*μ*, h^−1^) and lag phase duration (*λ*, h) of *L. monocytogenes* in raw fish are computed from the equations

$$\mu ={\left\{a_{Lm}\times {\left(Temp-T_{min}\right)}^{0.75}\right\}}^{2}$$

$$\lambda =exp\left(A_{Lm}\right)\times {\mu_{max}}^{{-m}_{Lm}}$$

where *T_min_* is the minimum growth temperature of *L. monocytogenes* in raw fish (1.3 °C) and *a_Lm_*, *A_Lm_*, and *m_Lm_* are empirical coefficients, set at 0.0581, 0.84, and 1.11, respectively [42]. In order to deal with the lag phase over multiple storage steps, the work to be “potentially” done at the current storage stage (*wk*) is calculated as

wk=μ×λ

and the work “actually” done is computed as

$${wk}_{actual}=wk-{WorkDone}_{s-1}$$

where *WorkDone_s-1_* is an input of the function and refers to the work done in a previous storage stage, *s-1*. At the first stage, *WorkDone_0_* takes the value of zero. The function updates the work done with the current expenditure of the lag phase, to produce *WorkDone_s_*

$${WorkDone}_{s}={WorkDone}_{s-1}+\mu \times time$$

where *WorkDone_s_* is an output that enables the reusability of the function over more than one storage stage, keeping track of the remaining lag phase. Next, the effective lag phase duration (*λ_eff_*) for the present storage stage is computed as

$$\lambda_{eff}=\left\{\begin{array}{c} 0,\;{wk}_{actual}<0\\ {wk}_{actual}/\mu ,\;{wk}_{actual}\geq 0,\;\mu >0\; \end{array}\right.$$

and deducted from *time* to produce the effective time (*time_eff_*) for loglinear growth,

$${time}_{eff}=\max\left(time-\lambda_{eff},0\right)$$

Considering *N_0_* (CFU) as the initial number of *L. monocytogenes* in a raw fish unit of weight *Unit_size_* grams, the number after storage (N, CFU) is estimated as

$$N=\left\{\begin{array}{c} 10^{\left(\log_{10}N_{0}+\frac{\mu }{ln(10)}{time}_{eff}\right)},if\;10^{\left(\log_{10}N_{0}+\frac{\mu }{ln(10)}{time}_{eff}\right)}<{Unit}_{size}\times 10^{MPD}\\ {Unit}_{size}\times 10^{MPD},if\;10^{\left(\log_{10}N_{0}+\frac{\mu }{ln(10)}{time}_{eff}\right)}\geq {Unit}_{size}\times 10^{MPD} \end{array}\right.$$

The inputs and outputs of the function sfGrowthLDP() are represented by

$$\left\{N,\;{WorkDone}_{t}\right\}=sfGrowthLPD\left(N_{0},\;time,\;Temp,\;{Unit}_{size},\;MPD,\;{WorkDone}_{s-1}\right)$$

### Appendix A.3. The Function sfRawFishStorage

The function sfGrowthLPD is applied to the contaminated fish units of the contamination matrix *N* (*N_ij_* > 0), setting the value of *WorkDone_t-1_* to zero,

$$\left\{N_{stored\;ij},\;{WorkDone}_{t\;ij}\right\}=sfGrowthLPD\left(N_{ij}\;,\;{time}_{Storage\;i},\;{Temp}_{Storage\;i},\;{Unit}_{size},\;MPD\right)\;j=1,\;2\ldots ,\;c$$

sfRawFishStorage() outputs are the contamination matrix after *L. monocytogenes* growth (*N_stored_*) and the work done after the first storage (*WorkDone_t_*, a matrix), and returns unaffected the mean prevalence of contaminated lots (*P*, a scalar) and the probability that the sampled lot is contaminated (*Prob_UnitPo_*_s_, a vector).

### Appendix A.4. The Function sfSlicer

The function starts by calculating the total number of slices or fillets (*nb_Slices_*) as

$${nb}_{Slices}=\left\lfloor \frac{{Unit}_{size}}{w_{Fillet}}\right\rfloor$$

Then, for each lot *i*, the transfer coefficients *a_i_* and *e_i_* are sampled from their variability distributions, whose values apply to all fish units produced in that lot $N_{i1},\;N_{i2},\;\ldots ,\;N_{ic}$,

$$a_{i}\;\sim \;Logistic\;\left({location}_{a},{scale}_{a}\right)\;0\leq a_{i}\leq 0.5$$

$${log}_{10}e_{i}\;\sim \;Normal\;\left(\mu_{loge},\sigma_{loge}\right)\;{log}_{10}e_{i}\leq 0$$

The parameters *location_a_*, *scale_a_*, *μ_loge_*, and *σ_loge_* are set to 0.07, 0.03, −2.12, and 0.85, respectively, according to Hoelzer et al. [43]. Following the assumption that the fish units present half the total contamination on one side, the transferable numbers of *L. monocytogenes* on the fish unit in the initial state 0 (*CFU_fish 0_*), before slicing, is obtained,

$${CFU}_{fish\;0\;ij}\;\sim \;Binomial\left(N_{stored\;ij},\;p=0.5\right)$$

and the bacterial numbers on the slicer blade in the initial state is set to *CFU_slicer 0_* (= *Init_Slicer_*). *Init_Slicer_* is assumed to be 1000 CFU. Then, computations to represent the *transition during slicing* are made, as follows [43]: for the *k*-th slice (*k* = 1, 2, …, *nb_Slices_* − 1), the *L. monocytogenes* cells from the fillet that are not lost are estimated:

$${M0}_{k\;ij}\;\sim \;Binomial\;\left({{CFU}_{fish\;0\;ij},\;1-e}_{i}\right)$$

The cells that remain on the slicer during the slicer-to-fish transfer (*M1_k_*) are estimated as

$${M1}_{k\;ij}\;\sim \;Binomial\;\left({CFU}_{slicer\;0\;ij},\;1-2a_{i}\right)$$

The cells that are transferred from the slicer to the fillet (*M2_k_*) are estimated as

$${M2}_{k\;ij}\;\sim \;Binomial\;\left({CFU}_{slicer\;0\;ij}-{M1}_{k\;ij},\;0.5\right)$$

The *L. monocytogenes* cells that are transferred from the slicer to the slice (*M3_k_*) are calculated as

$${M3}_{k\;ij}={CFU}_{slicer\;0\;ij}-{M1}_{k\;ij}-{M2}_{k\;ij}$$

The *L. monocytogenes* cells that are transferred from the fillet to the slicer are sampled from Binomial distributions (*S_k_*), as follows:

$$S_{k\;ij}\;\sim \;Binomial\;\left({M0}_{k\;ij},\;a_{j}\right)$$

Next, the computations linked to the *time step k after slicing* (*k*=1, 2, …, *nb_Slices_* − 1) are carried out,

$${CFU}_{slice\;\left(k+1\right)ij}={M3}_{k\;ij}+({M0}_{k\;ij}-S_{k\;ij})$$

$${CFU}_{slicer\;\left(k+1\right)ij}={M1}_{k\;ij}+S_{k\;ij}$$

$${CFU}_{fish\;\left(k+1\right)ij}={M2}_{k\;ij}$$

where ${CFU}_{slice\;\left(k+1\right)}$, ${CFU}_{slicer\;\left(k+1\right)}$, and ${CFU}_{fish\;\left(k+1\right)}$ are the numbers of *L. monocytogenes* on the slice, on the slicer blade, and on the fish unit, respectively, at time *k*. This procedure respects the mass balance, if one considers the $\left({CFU}_{fish\;0\;ij}-{M0}_{\;k\;ij}\right)\;$bacteria lost in the process [43]. This iterative algorithm continues slice by slice until reaching the last slice, *k* = *nb_Slices_*. At that point, it is assumed that the last slice takes the remaining contamination of the fish plus the remaining half of the original,

$${CFU}_{slice\;nbSlicesij}={CFU}_{fish\;\left(nbSlices-1\right)ij}+\left(N_{ij}-{CFU}_{fish\;0\;ij}\right)$$

*CFU_slice_* then becomes *N_Fillet_*, the numbers of *L. monocytogenes* on the fish fillets. It should be pointed out that, since one raw fish produces *nb_Slices_* fillets, the horizontal dimension of the contamination matrix *N_Fillet_* is enlarged, from *c* fish units to *c × nb_Slices_* fillet units. Thus, *N_Fillet_* is arranged to be a matrix of dimensions *r × c_f_*, where *c_f_ = c × nb_Slices_*. To keep track of the remaining microbial lag phase after the first storage of fish (output of the function sfRawFishStorage), the *r × c* matrix *WorkDone_t_* is also rearranged into a new matrix of dimensions *r × c_f_*, by attributing the value of *WorkDone_t_* of a fish unit to all of its *nb_Slices_* fillets.

Before returning the function outputs, there is a verification as to whether or not filleting renders any lot free of contamination (i.e., all the fillets produced in a lot having 0 CFU). This event, although infrequent, could occur in the case of low contamination of fish with the subsequent transfer of all cells to the slicer blade, or lost, and none to the fillets. In that case, the proportion of non-contaminated lots (*π*_0_) is estimated from the *N_Fillet_* matrix, and the mean prevalence value is corrected as

$$P_{Fillet}=P\times \left(1-\pi_{0}\right)$$

A value of one (1 CFU) is then ascribed in only one of the fillet units produced in the non-contaminated lots in order to avoid deletion of the “clean” lots and resampling of contaminated lots to round off the matrix size.

During the slicing of processed fish fillet, the function starts by calculating the total number of slices (*nb_Slices_*) as

$${nb}_{Slices}=\left\lfloor \frac{w_{Fillet}}{w_{Slice}}\right\rfloor$$

The algorithm proceeds exactly as during the filleting of raw fish. *CFU_slice_* becomes *N_Slice_,* the load of *L. monocytogenes* in processed fish slices. However, the horizontal dimension of the output contamination matrix *N_Slice_* is enlarged, from *c_f_* fillet units to *c_f_ × nb_Slices_* slices. *N_Fillet_* is now a matrix of dimensions *r × c_s_*, where *c_s_ = c_f_ × nb_Slices_*. In addition to *N_Slice_*, the function returns the probability vector of contaminated lots after filleting (*Prob_UnitPos Smoked_* for smoked fish slices or *Prob_UnitPos Gravad_* for gravad fish slices, in any case of unaltered values) and the mean prevalence of contaminated lots after slicing (*P_Slice_*).

### Appendix A.5. The Function sfBriningCC

The function sfBriningCC accounts for three possible scenarios [64]: (1) no cross-contamination occurring in lots already contaminated (*Lot_Pos__CC_Neg_*); (2) cross-contamination occurring in lots already contaminated (*Lot_Pos__CC_Pos_*); and (3) cross-contamination occurring in lots that were not contaminated (*Lot_Neg__CC_Pos_*). The probability of each scenario taking place for every lot *i* is calculated as

$$P\left({Lot}_{Pos}\_{CC}_{Neg\;i}\right)={Prob}_{UnitPosi}\times \left(1-{Pcc}_{brine}\right)$$

$$P\left({Lot}_{Pos}\_{CC}_{Pos\;i}\right)={Prob}_{UnitPosi}\times {Pcc}_{brine}$$

$$P\left({Lot}_{Neg}\_{CC}_{Pos\;i}\right)=\left(1-{Prob}_{UnitPosi}\right)\times {Pcc}_{brine}$$

The status of every lot (*I* = 1, 2,…, *r*) is then randomly sampled from the probabilities {$P\left({Lot}_{Pos}\_{CC}_{Neg\;i}\right),\;P\left({Lot}_{Pos}\_{CC}_{Pos\;i}\right),\;P\left({Lot}_{Neg}\_{CC}_{Pos\;i}\right)$}. This strategy is adopted in order to maintain the dimension [*r*, *c_f_*] of the input contamination matrix. At the same time, the volume (mL) of brine solution injected in a fish fillet (*volInj_i_*) and the concentration of *L. monocytogenes* (CFU/mL) in contaminated brine solution (*conc_Brine i_*) are sampled for every lot i from

$${volInj}_{i}\;\sim \;Pert\left({volInj}_{min},\;{volInj}_{mode},\;{volInj}_{max}\right)$$

$${Conc}_{Brine\;i}\;\sim \;Pert\left({Conc}_{Brine\;min},\;{Conc}_{Brine\;mode},\;{Conc}_{Brine\;max}\right)$$

where *volInj_min_, volInj_mode_*, and *volInj_max_* are, respectively, the minimum, most likely, and maximum parameters of the Pert distribution for brine volume injected, and *conc_Brine min_*, *conc_Brine mode_*, and *conc_Brine max_* are the minimum, most likely, and maximum parameters, respectively, of the Pert distribution for *L. monocytogenes* concentration in the salt solution. Values of 25, 35, and 100 mL for *volInj_min_, volInj_mode_*, and *volInj_max_* and 0, 0.0145, and 0.060 CFU/mL for *conc_Brine min_*, *conc_Brine mode_*, and *conc_Brine max_* were assumed as products of best guess.

Next, for each fillet, the potential number of cells transferred from the volume of brine (*N_transf_*) is obtained from

$$N_{transf\;ij}\;\sim \;Poisson\left({volInj}_{i}\times {Conc}_{Brine\;i}\right)\;j=1,\;2\ldots ,\;c_{f}$$

Next, depending on the status the sampled lot *i* takes, $N_{transf\;i\cdot }\;$is added to $N_{Hold\;i\cdot }$ (or not), in order to determine the numbers of *L. monocytogenes* in all fillets produced in lot *i* after brining (*N_Brine_*), according to

$$N_{Brine\;i\cdot }=\left\{\begin{array}{cc} N_{Hold\;i\cdot } & i\in {Lot}_{Pos}\_{CC}_{Neg}\\ N_{Hold\;i\cdot }+N_{transf\;i\cdot } & i\;\in \;{Lot}_{Pos}\_{CC}_{Pos}\\ N_{transf\;i\cdot } & i\in {Lot}_{Neg}\_{CC}_{Pos} \end{array}\right.$$

Finally, the probability vector of contaminated lots after brining, *Prob_UnitPos Brine_*, and the mean prevalence of contaminated lots after brining, *P_Brine_*, are updated, taking into account the probability that the brine solution is contaminated (*Pcc_Brine_*).

$${Prob}_{UnitPosBrinei}=1-\left(1-{Prob}_{UnitPosi}\right)\times \left(1-{Pcc}_{Brine}\right)$$

$$P_{Brine}=1-\left(1-P_{Hold}\right)\times \left(1-{Pcc}_{Brine}\right)$$

In summary, the inputs of the function sfBriningCC() are the outputs of sfRawFishStorage(), *Pcc_Brine_*, *volInj_min_*, *volInj_mode_*, *volInj_max_*, *conc_Brine min_*, *conc_Brine mode_*, and *conc_Brine max_*, whereas the outputs are the contamination matrix *N_Brine_*, the vector *Prob_UnitPos Brine_*, and the scalar *P_Brine_*.

### Appendix A.6. The Function sfSmearingCC

Since the contamination event is considered at the fillet level (probability of occurrence *Pcc_Smear_*), two scenarios must be taken into account: (1) the increase in contamination of the already contaminated lots, which is a scenario that produces lots of status ${Lot}_{Pos}\_CC$; and (2) the contamination of “clean” lots, which produces lots of status ${Lot}_{Neg}\_CC$. Each scenario has its own probability of occurrence.

To represent scenario 1, a contamination status matrix, *E_1_*, consisting of zeros (no contamination of the fish fillet during smearing) and ones (contamination of the fish fillet during smearing) is produced by evaluating the Bernoulli distribution at *Pcc_Smear_*,

$$E_{1ij}\;\sim \;Bernoulli\left({Pcc}_{Smear}\right)\;i=1,\;2\ldots ,\;r;\;j=1,\;2\ldots ,c_{f}$$

For the fish fillets *E_1 ij_* = 1, the load of *L. monocytogenes* cells (*N_TR1_*) transferred to a fish fillet is obtained by

$$N_{TR1\;ij}\;\sim \;Binomial\left(N_{surface},\;10^{{TR}_{Smear\;i}}\right)\;j=1,\;2,\;\ldots c_{f}$$

where the log_10_ of the transfer coefficient *TR_Smear i_* is sampled for every lot *i* from a normal distribution with mean $\mu_{TRsmear}=-0.29$ and $\sigma_{TRSmear}=0.31$ truncated on ]−∞, 0] [43]. A value of *N_surface_* of 100 CFU *L. monocytogenes* is best guessed.

$${TR}_{Smear\;i}\;\sim \;Normal\;\left(\mu_{TRsmear},\;\sigma_{TRSmear}\right),\;{TR}_{i}>0$$

Scenario 1 is finally represented by the sum of the initial contamination matrix, *N_Hold_*, and the matrix of the newly contaminated fish fillets, *N_TR1_*.

$$N_{1\;ij}=N_{Hold\;ij}+N_{TR1\;ij}$$

To represent scenario 2, a matrix of zeros *N*_0_ (i.e., non-contaminated fish fillets) of dimensions $r_{2}=\left\lceil r\times (\frac{1}{P}-1)\right\rceil$ and $c_{2}=c_{f}$ is first produced, and the same microbial transfer simulation on fish fillets, as explained before, is performed,

$$E_{2ij}\;\sim \;Bernoulli\left({Pcc}_{Smear}\right)\;i=1,\;2\ldots ,\;r_{2};\;j=1,\;2\ldots ,c_{2}$$

$${TR}_{Smear\;i}\;\sim \;Normal\;\left(-0.29,\;0.31\right),\;{TR}_{i}>0$$

$$N_{TR2\;ij}\;\sim \;Binomial\left(N_{surface},\;10^{{TR}_{Smear\;i}}\right)\;j=1,\;2,\;\ldots c_{2}$$

Scenario 2 is finally characterized by the sum of the “clean lots” matrix, *N*_0_, and the matrix of the newly contaminated fish fillets, *N_TR2_*.

$$N_{2\;ij}=N_{0\;ij}+N_{TR2\;ij}$$

However, it is possible that lots of fillets remain non-contaminated in the matrix *N_2_* (rows with zero counts). Those lots are identified and removed from *N*_2_, and the proportion of clean (removed) lots estimated ($\pi_{0N2}$).

So far, two contamination matrices of different numbers of lots have been produced, *N_1_* and *N_2_*, which occur at the probabilities *P*_1_ and *P*_2_, respectively.

$$P_{1}=P_{Hold}$$

$$P_{2}=\left(1-P_{Hold}\right)\times \left(1-\pi_{0N2}\right)$$

For the function to return a joint contamination matrix output (*N_Smear_*) of the same original size (*r*, *c_f_*), the status of every lot, *i* {${Lot}_{Pos}\_CC$, ${Lot}_{Neg}\_CC\}$ (i=1, 2, …., r), is then randomly sampled from the probabilities {$P_{1},\;P_{2}$}.

$$N_{Smear\;i\cdot }=\left\{\begin{array}{c} N_{1\;i\cdot }\;i\in {Lot}_{Pos}\_CC\\ N_{2\;i\cdot \;}\;i\;\in {Lot}_{Neg}\_CC \end{array}\right.\;i=1,\;2,\;\ldots ,\;c_{f}$$

The resulting matrix *N_Smear_* contains the numbers of *L. monocytogenes* cells in *c_f_* fish fillets (horizontal dimension) after a contamination event during smearing in a sample of *r* contaminated lots (vertical dimension). Finally, the probability vector of contaminated lots after smearing, *Prob_UnitPos Smear_*, and the mean prevalence of contaminated lots after smearing, *P_Smear_*, are computed, as follows:

$${Prob}_{UnitPosSmeari}={Prob}_{UnitPosi}+\left(1-{Prob}_{UnitPosi}\right)\times \left(1-\pi_{0N2}\right)$$

$$P_{Smear}=P_{1}+P_{2}$$

Summarizing, the function sfSmearingCC() is fed by the outputs of sfRawFishStorage(), *Pcc_Smear_*, $N_{surface}$, *μ_TRSmear_*, and *σ_TRSmear_*, whereas the outputs are the contamination matrix *N_Smear_*, the vector *Prob_UnitPos Smear_*, and the scalar *P_Smear_*.

### Appendix A.7. The Function sfBrineORsaltCC

The functions commence by constructing the lot status vector *Type* to assign the type of salting that will be applied to every lot *i* of the contamination matrix *N_Hold_*. *Type* is coded 1 for brining and 0 for dry-salting,

$${Type}_{i}\;\sim \;Bernoulli\;\left(p_{Brine}\right)\;i=1,\;2,\;\ldots ,\;r$$

The input contamination matrix *N_Hold_* is partitioned into two contamination matrices (*N_Hold_*_1_ and *N_Hold_*_0_), according to the type of salting. The same is carried out for the probability vector *Prob_UnitPos_* to produce two vectors, *Prob_UnitPos_*_1_ and *Prob_UnitPos_*_0_.

$$N_{Hold1}=N_{Hold\;i\cdot }\;\forall \;i\;{|\;Type}_{i}=1$$

$$N_{Hold0}=N_{Hold\;i\cdot }\;\forall \;i\;{|\;Type}_{i}=0$$

$${Prob}_{UnitPos\;1}={Prob}_{UnitPos\;i}\;\forall \;i\;{|\;Type}_{i}=1\;{Prob}_{UnitPos\;0}={Prob}_{UnitPos\;i}\;\forall \;i\;{|\;Type}_{i}=0$$

The auxiliary functions are then applied, according to the type of salting,

$${[N}_{Brine},\;{Prob}_{UnitPosBrine}]=sfBriningCC\left(\begin{array}{c} N_{Hold\;1},\;{Prob}_{UnitPos\;1},\;P_{Hold},\;{Pcc}_{Smear},N_{surface},\;\\ {volInj}_{min},{volInj}_{mode},\;{volInj}_{max},{Conc}_{Brine\;min},\\ \;{Conc}_{Brine\;mode},\;{Conc}_{Brine\;max} \end{array}\right)$$

$${[N}_{Smear},\;{Prob}_{UnitPosSmear}]=sfSmearingCC\left(\begin{array}{c} N_{Hold\;0},\;{Prob}_{UnitPos\;0},\;P_{Hold},\;{Pcc}_{brine},N_{surface},\\ \mu_{TRsmear},\;\sigma_{TRSmear} \end{array}\right)$$

The contamination matrix after brining or salting, *N_BrineORSalt_*, is arranged from the previous outputs, maintaining the index locations of *Type*,

$$N_{BrineORSalt\;i}=\left\{\begin{array}{c} N_{Brine\;i\cdot }\;\;\forall \;i\;{|\;Type}_{i}=1\\ N_{Smear\;i\cdot }\;\;\forall \;i\;{|\;Type}_{i}=0 \end{array}\right.\;i=1,\;2,\;\ldots ,\;r$$

In the same way, the probability vector of contaminated lots after brining or salting (*Prob_UnitPos BrineORSalt_*) is then arranged from the *Prob_UnitPos_* outputs of the auxiliary functions, correcting them by *p_Brine_*.

$${Prob}_{UnitPos\;BrineORSalt\;i}=\left\{\begin{array}{c} {Prob}_{UnitPos\;Brine\;i}\times p_{Brine}\;\forall \;i\;{|\;Type}_{i}=1\\ {Prob}_{UnitPos\;Smear\;i}\times \left(1-p_{Brine}\right)\;\forall \;i\;{|\;Type}_{i}=0 \end{array}\right.\;i=1,\;2,\;\ldots ,\;r$$

The mean prevalence of contaminated lots after brining or salting (*P_BrineORSalt_*) is computed as

$$P_{BrineORSalt}=1-\left\{p_{Brine}\times \left(1-P_{Hold}\right)\times \left(1-{Pcc}_{Brine}\right)\;+\left(1-p_{Brine}\right)\times \left(1-P_{Hold}\right)\times \left(1-{Pcc}_{Smear}\right)\right\}$$

The information contained in the vector *Type* is to be passed to the function sfSmoking(), since the extent of microbial reduction during smoking depends on the type of salting.

The inputs of the function sfBrineORsaltCC() are the probability of brining (*p_Brine_*) and the inputs of the auxiliary functions sfBriningCC and/or sfSmearingCC, whereas the outputs are the contamination matrix *N_BrineORSalt_*, the probability of contaminated lots (*Prob_UnitPos BrineORSalt_*, a vector), the mean prevalence of contamination (*P_BrineORSalt_*, a scalar), and the type of salting of the lots (*Type*, a vector).

### Appendix A.8. The Function sfSmoking

The algorithm starts by sampling the extent of microbial log_10_ reduction *R_Brine i_* or *R_Drysalt i_* for every lot *i*, conditional to the type of salting the fish fillets of that lot were subjected to. This information is stored in r-length vector *Type*.

$$R_{Brine\;i}\;\sim \;Normal\left(\mu_{RBrine},\;\sigma_{RBrine}\right)\;R_{Brine\;i}>0,\;\forall \;i\;|\;{Type}_{i}=“1”$$

$$R_{Drysalt\;i}\;\sim \;Normal\left(\mu_{RDrysalt},\;\sigma_{RDrysalt}\right)\;R_{Brine\;i}>0,\;\forall \;i\;|\;{Type}_{i}=“0”$$

For every lot *i*, the probability of survival of a microbial cell, *p_Survive i_*, is computed as

$$p_{Survive\;i}=\left\{\begin{array}{c} 10^{R_{Brine\;i}}\;\;\forall \;i\;|\;{Type}_{i}=“1”\\ 10^{R_{Dry\;salt\;i}}\;\;\forall \;i\;|\;{Type}_{i}=“0” \end{array}\right.$$

and the number of surviving cells after smoking, *N_Smoked_*, is obtained by

$$N_{Smoked\;ij}\;\sim \;Binomial\left(N_{BrineORSalt\;ij}\;,p_{Survive\;i}\;\right)\;j=1,\;2,\;\ldots ,\;c_{f}$$

To update the prevalence estimates after smoking and maturation, the probability of at least one cell surviving in the lot *i* (*atLeastOne_i_*) is calculated:

$${atLeastOne}_{i}=1-{\left(1-p_{Survive\;i}\right)}^{\sum_{j=1}^{cf}N_{BrineORSalt\;ij}}$$

The probability vector of contaminated lots after smoking and maturation, *Prob_UnitPos Smoked_*, and the mean prevalence of contaminated lots, *P_Smoked_*, are then updated,

$${Prob}_{UnitPosSmokedi}={Prob}_{UnitPos\;BrineORSalti}\times {atLeastOne}_{i}$$

$$P_{Smoked}=P_{BrineORSalt}\times \frac{\sum_{i=1}^{r}{atLeastOne}_{i}}{r}$$

The function sfSmoking() returns the prevalences *Prob_UnitPos Smoked_*, *P_Smoked_*, and the contamination matrix *N_Smoked_* containing the load of *L. monocytogenes* in the fish fillets originated from the contaminated lots.

### Appendix A.9. The Function sfMaceration

The algorithm starts by estimating the probability of survival of a microbial cell, *p_Survive i_*, in every lot *i*,

$$R_{Gravad\;i}\;\sim \;Normal\left(\mu_{RGravad},\;\sigma_{RGravad}\right)\;R_{Gravad\;i}>0$$

$$p_{Survive\;i}=10^{-R_{Gravad\;i}}$$

and the number of surviving cells after maceration or curing, *N_Macerated_*, is obtained by

$$N_{Gravad\;ij}\;\sim \;Binomial\left(N_{Smear\;ij}\;,p_{Survive\;i}\;\right)\;j=1,\;2,\;\ldots ,\;c_{f}$$

The probability of at least one cell surviving in the lot *i* (*atLeastOne_i_*) is calculated as

$${atLeastOne}_{i}=1-{\left(1-p_{Survive\;i}\right)}^{\sum_{j=1}^{cf}N_{Smear\;ij}}$$

The probability vector of contaminated lots after maceration, *Prob_UnitPos Gravad_*, and the mean prevalence of contaminated lots, *P_Gravad_*, are then updated,

$${Prob}_{UnitPosGravad\;i}={Prob}_{UnitPos\;Smeari}\times {atLeastOne}_{i}$$

$$P_{Gravad}=P_{Smear}\times \frac{\sum_{i=1}^{r}{atLeastOne}_{i}}{r}$$

The function sfMaceration() returns the contamination matrix *N_Gravad_* containing the load of *L. monocytogenes* in gravad fish fillets, the lot-specific probability of contaminated gravad fish fillets (*Prob_UnitPos Gravad_*, a vector), and the mean prevalence of contaminated gravad fish fillets (*P_Gravad_*, a scalar).

### Appendix A.10. The Function sfTesting

The function sfTesting() receives the outputs of the function sfPackaging(): *n*, the number of tested units; *g*, the sub-sample weight in grams used for detection; *c*, the number of positive samples accepted (two-class or three-class mixed plan); *M*, the maximum limit concentration; *p_lot tested_*, the proportion of tested lots; *Se*, the probability of the test (enumeration or detection) to detect, independently, each bacteria present in a sample; and *g_TestedEnum_*, the sub-sample weight in grams used for the enumeration assay.

Assuming perfect homogenization of the sample, each of the bacterium present in each of the *r* × *c_p_* units of *Unit_SizePack_* weight has a probability of being present in the *g* grams of the sub-sample *and* detected equal to $Se\times {g/Unit}_{SizePack}$. The number of bacteria detected in the detection assay is then

$$N_{Detected\;ij}\;\sim \;Binomial\;\left(N_{Pack\;ij}\;,Se\times {g/Unit}_{SizePack}\right)$$

and the detection test is positive if $N_{Detected\;ij}>0$.

If the sampling plan is a three-class plan, an enumeration test (direct plating) is performed. The number of bacteria enumerated in the sample is

$$N_{Enumerated\;ij}\;\sim \;Binomial\;\left(N_{Pack\;ij}\;,Se\times {g_{TestedEnum}/Unit}_{SizePack}\;\right)$$

and the estimated concentration is $N_{Enumerated\;ij}/g_{TestedEnum}$ CFU/g. The algorithm assumes that the enumeration is performed only on samples positive in detection.

In order to evaluate the probability of each lot to be rejected, 1000 (by default) Monte Carlo samples of *n* samples are constituted for each lot and, for each of these Monte Carlo samples, the microbiological criteria is applied (i.e., in a two-class plan, the lot is rejected if >*c* samples among *n* are detected, whereas in a three-class plan the lot is rejected if >*c* samples among *n* are detected or if at least one sample has an estimated concentration > *M* CFU/g). The mean number of times the lot is rejected among the Monte Carlo samples multiplied by the probability for the lot to be tested is an estimate of *P_pos i_*, the probability of lot *i* being rejected.

Given the probability of lot *i* being contaminated pre-testing (*Prob_UnitPos_*, which corresponds to *Prob_UnitPos Smoked_* for smoked fish slices, or to *Prob_UnitPos Gravad_* for gravad fish slices) and the probability for lot *i* to be rejected (*P_pos i_*), the prevalence of contaminated lots given that they were not detected after testing (*Prob_UnitPos tested i_*) is

$${Prob}_{UnitPos\;tested\;i}=\frac{\left(1-P_{pos\;i}\right)\times {Prob}_{UnitPos\;i}}{\left(1-P_{pos\;i}\right)\times {Prob}_{UnitPos\;i}+\left(1-{Prob}_{UnitPos\;i}\right)}$$

The mean prevalence of contaminated lots after within-lot testing (*P_Tested_*, a scalar) is therefore

$$P_{Tested}=\frac{\left(1-\frac{\sum_{i=1}^{r}P_{pos\;i}}{r}\right)\times P_{Pack}}{\left(1-\frac{\sum_{i=1}^{r}P_{pos\;i}}{r}\right)\times P_{Pack}+\left(1-P_{Pack}\right)}$$

### Appendix A.11. The Function sfMejlholmDalgaard

The growth rate of *L. monocytogenes* ($\mu_{LM}$ [h^−1^]) in RTE seafood is calculated as

$$\mu_{LM}=\mu_{LM\;ref}\times \gamma_{T}\left(T\right)\times \gamma_{a_{w}}\left(a_{w}\right)\times \gamma_{pH}\left(pH\right)\times \gamma_{LAC}\left(\left[LAC\right]\right)\times \gamma_{Phe}\left(phe\right)\times \gamma_{nit}\left(nit\right)\;\times \gamma_{CO2}\left(\left[CO2\right]\right)\times \gamma_{DAC}\left(\left[DAC\right]\right)\times \gamma_{AAC}\left(\left[AAC\right]\right)\times \gamma_{BAC}\left(\left[BAC\right]\right)\times \gamma_{CAC}\left(\left[CAC\right]\right)\times \gamma_{SAC}\left(\left[SAC\right]\right)\;\times {\xi \left(T,a_{w},pH,\left[LAC\right],phe,nit,\left[CO2\right],\left[DAC\right],\left[AAC\right],\left[BAC\right],\left[CAC\right],[SAC]\right)}_{int}$$

where $\mu_{LM\;ref}$ (=0.419 h^−1^) is the optimum growth rate of *L. monocytogenes* in the RTE seafood at the reference temperature (*T_ref_*) of 25 °C, $\gamma_{x}(x)$ are the cardinal terms for the independent effect of the environmental parameter *x*, and ξ() is the term describing the effect of interactions between environmental parameters. Each of the terms are calculated as follows:

$$\gamma_{T}\left(T\right)=\left\{\begin{array}{cc} {\left(\frac{T-(-2.83)}{25-(-2.83)}\right)}^{2} & if\;T>-2.83{}^{\circ}C\\ 0 & if\;T\leq -2.83{}^{\circ}C \end{array}\right.$$

where *T* is the temperature in °C. The value of −2.83 belongs to *L. monocytogenes* minimum temperature for growth [53].

$$\gamma_{a_{w}}\left(a_{w}\right)=\left\{\begin{array}{cc} \frac{\left(a_{w}-0.923\right)}{\left(1-0.923\right)} & if\;a_{w}>0.923\\ 0 & if\;a_{w}\leq 0.923 \end{array}\right.$$

where *a_w_* is the water activity of the RTE seafood product. The minimum *a_w_* for *L. monocytogenes* growth is 0.923 [53]. The value of *a_w_* can be estimated from the percentage of salt content [NaCl] [%].

$$a_{w}=0.999489-0.005179\times \left[NaCl\right]-0.0001272\times {\left[NaCl\right]}^{2}$$

$$\gamma_{pH}\left(pH\right)=\left\{\begin{array}{cc} 1-10^{\left(4.97-pH\right)} & if\;pH>4.97\\ 0 & if\;pH\leq 4.97 \end{array}\right.$$

where pH is the pH of the RTE seafood product. The value of 4.97 corresponds to *L. monocytogenes* minimum pH for growth [53].

$$\gamma_{LAC}\left(\left[LAC_{U}\right]\right)=\left\{\begin{array}{cc} \left(1-\frac{\left[LAC_{U}\right]}{3.79}\right) & if\;\left[LAC_{U}\right]<3.79\;mM\\ 0 & f\;\left[LAC_{U}\right]\geq 3.79\;mM \end{array}\right.$$

where [*LAC_U_*] is the concentration (mM) of undissociated lactic acid and the value of 3.79 belongs to the minimum inhibitory concentration (MIC) of *LAC_U_*. [*LAC_U_*] can be estimated from the concentration of total lactic acid (*LA_tot_*) in ppm,

$$\left[LAC_{U}\right]=\frac{{LA}_{tot}}{90.08\times \left(1+10^{pH-3.86}\right)}$$

$$\gamma_{Phe}\left(Phe\right)=\left\{\begin{array}{cc} \left(\frac{32-Phe}{32}\right) & if\;PHE<32\;ppm\\ 0 & if\;PHE\geq 32\;ppm \end{array}\right.$$

where *Phe* is the concentration of phenol compound (ppm). The value of 32 ppm belongs to the maximum *Phe* for *L. monocytogenes* growth [53].

$$\gamma_{nit}\left(nit\right)=\left\{\begin{array}{cc} {\left(\frac{350-nit}{350}\right)}^{2} & if\;nit<350\;ppm\\ 0 & if\;nit\geq 350\;ppm \end{array}\right.$$

where *nit* is the concentration of nitrites (ppm). The maximum *nit* for *L. monocytogenes* growth is 350 ppm [53].

$$\gamma_{CO2}\left(\left[CO2\right]\right)=\left\{\begin{array}{cc} \left(\frac{3140-\left[CO2\right]}{3140}\right) & if\;\left[CO2\right]<3140\;mM\\ 0 & if\;\left[CO\right]\geq 3140\;mM \end{array}\right.$$

where [*CO*2] is the concentration of CO*_2_* in mM, and the value of 3140 mM belongs to the maximum [*CO*2] for *L. monocytogenes* growth [53]. The value of [*CO*2] can be estimated from the CO_2_ concentration at equilibrium in % (${CO2}_{equi}$),

$$\left[CO2\right]=\frac{{CO2}_{equi}\times 101,323\times 2.4429}{\exp\left(-6.8346+\frac{12,817}{T+273.15}-\frac{3,766,800}{{\left(T+273.15\right)}^{2}}+\frac{2.997E8}{{\left(T+273.15\right)}^{3}}\right)}$$

$$\gamma_{DAC}\left(\left[DAC_{U}\right]\right)=\left\{\begin{array}{cc} \left(1-\sqrt{\frac{\left[DAC_{U}\right]}{4.8}}\right) & if\;\left[DAC_{U}\right]<4.8\;mM\\ 0 & f\;\left[DAC_{U}\right]\geq 4.8\;mM \end{array}\right.$$

where [*DAC_U_*] is the concentration of undissociated diacetate (mM). The minimum inhibitory concentration of *DAC_U_* is 4.8 mM [53]. [*DAC_U_*] can be estimated from the total diacetate ${DA}_{tot}$ (in ppm),

$$\left[DAC_{U}\right]=\frac{{DA}_{tot}}{119.1\times \left(1+10^{pH-4.76}\right)}$$

$$\gamma_{AAC}\left(\left[AAC_{U}\right]\right)=\left\{\begin{array}{cc} \left(1-\sqrt{\frac{\left[AAC_{U}\right]}{10.3}}\right) & if\;\left[AAC_{U}\right]<10.3\;mM\\ 0 & if\;\left[AAC_{U}\right]\geq 10.3\;mM \end{array}\right.$$

where [*AAC_U_*] is the concentration of undissociated acetic acid (mM). The value of 10.3 mM corresponds to the minimum inhibitory concentration of *AAC_U_* [53]. [*AAC_U_*] can be estimated from the total acetic acid (${AA}_{tot}$) in ppm,

$$\left[AAC_{U}\right]=\frac{{AA}_{tot}}{60.05\times \left(1+10^{pH-4.76}\right)}$$

$$\gamma_{BAC}\left(\left[BAC_{U}\right]\right)=\left\{\begin{array}{cc} \left(1-\frac{\left[BAC_{U}\right]}{0.349}\right) & if\;\left[BAC_{U}\right]<0.349\;mM\\ 0 & if\;\left[BAC_{U}\right]\geq 0.349\;mM \end{array}\right.$$

where [*BAC_U_*] is the concentration of undissociated benzoic acid (mM). The minimum inhibitory concentration of *BAC_U_* is 0.349 mM [53]. [*BAC_U_*] can be estimated from the total BA (${BA}_{tot}$) in ppm,

$$\left[BAC_{U}\right]=\frac{{BA}_{tot}}{122.12\times \left(1+10^{pH-4.19}\right)}$$

$$\gamma_{CAC}\left(\left[CAC_{U}\right]\right)=\left\{\begin{array}{cc} \left(1-\frac{\left[CAC_{U}\right]}{2.119}\right) & if\;\left[CAC_{U}\right]<2.119\;mM\\ 0 & if\;\left[CAC_{U}\right]\geq 2.119\;mM \end{array}\right.$$

where [*CAC_U_*] is the concentration of undissociated citric acid (mM). The minimum inhibitory concentration of *CAC_U_* is 2.119 mM [53]. [*CAC_U_*] can be computed from the total CA (${CA}_{tot}$) in ppm,

$$\left[CAC_{U}\right]=\frac{{CA}_{tot}}{192.13\times \left(1+10^{pH-3.13}\right)}$$

$$\gamma_{SAC}\left(\left[SAC_{U}\right]\right)=\left\{\begin{array}{cc} \left(1-\frac{\left[SAC_{U}\right]}{1.896}\right) & if\;\left[SAC_{U}\right]<1.896\;mM\\ 0 & if\;\left[SAC_{U}\right]\geq 1.896\;mM \end{array}\right.$$

where [*SAC_U_*] is the concentration of undissociated sorbic acid (mM). The minimum inhibitory concentration of *SAC_U_* is 1.896 mM [53]. $\left[SAC_{U}\right]$ can be estimated from the total SA (${SA}_{tot})$ in ppm,

$$\left[SAC_{U}\right]=\frac{{SA}_{tot}}{112.1\times \left(1+10^{pH-4.76}\right)}$$

As for the interaction term, Mejlholm and Dalgaard [52] use the Le Marc [77] approach,

$$\xi \{\phi \left(T,a_{w},pH,nit,phe,\;CO2,acids\right)\}=\left\{\begin{array}{cc} 1 & if\;\psi \leq 0.5\\ 2\left(1-\psi \right) & if\;0.5<\psi <1\\ 0 & if\;\psi \geq 1 \end{array}\right.\;\;$$

where

$$\;\psi =\sum_{i}\left(\frac{\phi \left(i\right)}{2\prod_{j\neq i}\left(1-\phi \left(j\right)\right)}\right)$$

and

$$\phi \left(T\right)={\left(1-\frac{T+2.83}{25+2.83}\right)}^{2}\;\phi \left(a_{w}\right)={\left(1-\sqrt{\frac{a_{w}-0.923}{1-0.923}}\right)}^{2}\;\phi \left(pH\right)={\left(1-\sqrt{1-10^{\left(4.97-pH\right)}}\right)}^{2}\;\phi \left(phe\right)={\left(1-\sqrt{\frac{32-Phe}{32}}\right)}^{2}\;\phi \left(nit\right)={\left(1-\frac{350-nit}{350}\right)}^{2}\;\phi \left(CO2\right)={\left(1-\sqrt{\frac{3140-CO2}{3140}}\right)}^{2}\;$$

$$\phi \left(acids\right)={\left(1-\left(1-\sqrt{\frac{\left[LAC_{U}\right]}{3.79}}\right)\times \left(1-\sqrt{\frac{\left[DAC_{U}\right]}{4.80}}\right)\times \left(1-\sqrt{\frac{\left[AAC_{U}\right]}{10.3}}\right)\;\times \left(1-\frac{\left[BAC_{U}\right]}{0.349}\right)\times \left(1-\frac{\left[CAC_{U}\right]}{2.119}\right)\times \left(1-\frac{\left[SAC_{U}\right]}{1.896}\right)\right)}^{2}$$

### Appendix A.12. The Function sfMejlholmDalgaardLAB

The growth rate of lactic acid bacteria ($\mu_{LAB}$ [h^−1^]) in RTE seafood is computed as,

$$\mu_{LAB}=\mu_{LAB\;ref}\times \gamma_{T}\left(T\right)\times \gamma_{a_{w}}\left(a_{w}\right)\times \gamma_{pH}\left(pH\right)\times \gamma_{LAC}\left(\left[LAC\right]\right)\times \gamma_{Phe}\left(phe\right)\times \gamma_{nit}\left(nit\right)\;\gamma_{CO2}\left(\left[CO2\right]\right)\times \gamma_{DAC}\left(\left[DAC\right]\right)\times \gamma_{AAC}\left(\left[AAC\right]\right)\times \gamma_{BAC}\left(\left[BAC\right]\right)\times \gamma_{CAC}\left(\left[CAC\right]\right)\times \gamma_{SAC}\left(\left[SAC\right]\right)\;\times {\xi \left(T,a_{w},pH,\left[LAC\right],phe,nit,\left[CO2\right],\left[DAC\right],\left[AAC\right],\left[BAC\right],\left[CAC\right],[SAC]\right)}_{int}$$

where $\mu_{LAB\;ref}$ (=0.583 h^−1^) is the optimum growth rate of LAB in the RTE seafood at the reference temperature (*T_ref_*) of 25 °C. Each of the cardinal terms, $\gamma_{x}(x)$, and the interaction, ξ(), are calculated in a similar way as for the *L. monocytogenes* growth rate equation, yet with cardinal parameters specific for LAB [54].

$$\gamma_{T}\left(T\right)=\left\{\begin{array}{cc} {\left(\frac{T-(-5.25)}{25-(-5.25)}\right)}^{2} & if\;T>-5.25\;{}^{\circ}C\\ 0 & if\;T\leq -5.25\;{}^{\circ}C \end{array}\right.$$

where *T* is the temperature in °C.

$$\gamma_{a_{w}}\left(a_{w}\right)=\left\{\begin{array}{cc} \frac{\left(a_{w}-0.928\right)}{\left(1-0.928\right)} & if\;a_{w}>0.928\\ 0 & if\;a_{w}\leq 0.928 \end{array}\right.$$

where a_w_ is the water activity of the RTE seafood product.

$$\gamma_{pH}\left(pH\right)=\left\{\begin{array}{cc} 1-10^{\left(4.24-pH\right)} & if\;pH>4.24\\ 0 & if\;pH\leq 4.24 \end{array}\right.$$

where pH is the pH of the RTE seafood product.

$$\gamma_{LAC}\left(\left[LAC_{U}\right]\right)=\left\{\begin{array}{cc} \left(1-\frac{\left[LAC_{U}\right]}{12}\right) & if\;\left[LAC_{U}\right]<12\;mM\\ 0 & f\;\left[LAC_{U}\right]\geq 12\;mM \end{array}\right.$$

where [*LAC_U_*] is the concentration (mM) of undissociated lactic acid.

$$\gamma_{Phe}\left(Phe\right)=\left\{\begin{array}{cc} \left(\frac{40.3-Phe}{40.3}\right) & if\;PHE<40.3\;ppm\\ 0 & if\;PHE\geq 40.3\;ppm \end{array}\right.$$

where *Phe* is the concentration of phenol compound (ppm).

$$\gamma_{nit}\left(nit\right)=\left\{\begin{array}{cc} {\left(\frac{2780-nit}{2780}\right)}^{2} & if\;nit<2780\;ppm\\ 0 & if\;nit\geq 2780\;ppm \end{array}\right.$$

where *nit* is the concentration of nitrites (ppm).

$$\gamma_{CO2}\left(\left[CO2\right]\right)=\left\{\begin{array}{cc} \left(\frac{6691-\left[CO2\right]}{6691}\right) & if\;\left[CO2\right]<6691\;mM\\ 0 & if\;\left[CO\right]\geq 6691\;mM \end{array}\right.$$

where *[CO2]* is the concentration of CO*_2_* in mM.

$$\gamma_{DAC}\left(\left[DAC_{U}\right]\right)=\left\{\begin{array}{cc} \left(1-\sqrt{\frac{\left[DAC_{U}\right]}{33.3}}\right) & if\;\left[DAC_{U}\right]<33.3\;mM\\ 0 & f\;\left[DAC_{U}\right]\geq 33.3\;mM \end{array}\right.$$

where [*DAC_U_*] is the concentration of undissociated diacetate (mM).

$$\gamma_{AAC}\left(\left[AAC_{U}\right]\right)=\left\{\begin{array}{cc} \left(1-\sqrt{\frac{\left[AAC_{U}\right]}{10.3}}\right) & if\;\left[AAC_{U}\right]<10.3\;mM\\ 0 & if\;\left[AAC_{U}\right]\geq 10.3\;mM \end{array}\right.$$

where [*AAC_U_*] is the concentration of undissociated acetic acid (mM).

$$\gamma_{BAC}\left(\left[BAC_{U}\right]\right)=\left\{\begin{array}{cc} {\left(1-\frac{\left[BAC_{U}\right]}{1.51}\right)}^{2} & if\;\left[BAC_{U}\right]<1.51\;mM\\ 0 & if\;\left[BAC_{U}\right]\geq 1.51\;mM \end{array}\right.$$

where [*BAC_U_*] is the concentration of undissociated benzoic acid (mM).

$$\gamma_{CAC}\left(\left[CAC_{U}\right]\right)=\left\{\begin{array}{cc} {\left(1-\frac{\left[CAC_{U}\right]}{10.3}\right)}^{2} & if\;\left[CAC_{U}\right]<10.3\;mM\\ 0 & if\;\left[CAC_{U}\right]\geq 10.3\;mM \end{array}\right.$$

where [*CAC_U_*] is the concentration of undissociated citric acid (mM).

$$\gamma_{SAC}\left(\left[SAC_{U}\right]\right)=\left\{\begin{array}{cc} {\left(1-\frac{\left[SAC_{U}\right]}{12.6}\right)}^{2} & if\;\left[SAC_{U}\right]<12.6\;mM\\ 0 & if\;\left[SAC_{U}\right]\geq 12.6\;mM \end{array}\right.$$

where [*SAC_U_*] is the concentration of undissociated sorbic acid (mM).

The interaction term is determined as

$$\xi \{\phi \left(T,a_{w},pH,nit,phe,\;CO2,acids\right)\}=\left\{\begin{array}{cc} 1 & if\;\psi \leq 0.5\\ 2\left(1-\psi \right) & if\;0.5<\psi <1\\ 0 & if\;\psi \geq 1 \end{array}\right.\;\;$$

where

$$\;\psi =\sum_{i}\left(\frac{\phi \left(i\right)}{2\prod_{j\neq i}\left(1-\phi \left(j\right)\right)}\right)$$

and

$$\phi \left(T\right)={\left(1-\frac{T+5.25}{25+5.25}\right)}^{2}\;\phi \left(a_{w}\right)={\left(1-\sqrt{\frac{a_{w}-0.928}{1-0.928}}\right)}^{2}\;\phi \left(pH\right)={\left(1-\sqrt{1-10^{\left(4.24-pH\right)}}\right)}^{2}\;\phi \left(phe\right)={\left(1-\sqrt{\frac{40.3-Phe}{40.3}}\right)}^{2}\;\phi \left(nit\right)={\left(1-\frac{2780-nit}{2780}\right)}^{2}\;\phi \left(CO2\right)={\left(1-\sqrt{\frac{6691-CO2}{6691}}\right)}^{2}\;$$

$$\phi \left(acids\right)={\left(1-\left(1-\sqrt{\frac{\left[LAC_{U}\right]}{12}}\right)\times \left(1-\sqrt{\frac{\left[DAC_{U}\right]}{33.3}}\right)\times \left(1-\sqrt{\frac{\left[AAC_{U}\right]}{10.3}}\right)\;\times \left(1-\frac{\left[BAC_{U}\right]}{1.51}\right)\times \left(1-\frac{\left[CAC_{U}\right]}{10.3}\right)\times \left(1-\frac{\left[SAC_{U}\right]}{12.6}\right)\right)}^{2}$$

### Appendix A.13. The Function sfGrowthJameson

The inputs of sfGrowthJameson() are the time (*t* in h), the initial numbers of *L. monocytogenes* and LAB in the food unit (*N_0 LM_*, *N_0 LAB_* in CFU), the initial values of the ideal substance for *L. monocytogenes* and LAB (*q_0 LM_*, *q_0 LAB_*), the maximum population densities of *L. monocytogenes* and LAB (*MPD_LM_*, *MPD_LAB_* in log_10_ CFU/g), the food unit’s weight (*Unit_Size_* in g), and the growth rates of *L. monocytogenes* and LAB ($\mu_{LM}$ and $\mu_{LAB}$ in h^−1^), as determined by the functions sfMejlholmDalgaard() and sfMejlholmDalgaardLAB(), respectively.

The following system of differential equations is integrated from time = 0 to time = *t*,

$$\frac{1}{N_{LM}(t)}\times \frac{dN_{LM}(t)}{dt}={\left(\frac{q_{LM}(t)}{1+q_{LM}(t)}\right)\times \mu }_{LM}\times \left(1-\frac{N_{LM}}{{{Unit}_{Size}\times 10}^{{MPD}_{LM}}}\right)\times \left(1-\frac{{\gamma \times N}_{LAB}}{{{Unit}_{Size}\times 10}^{{MPD}_{LAB}}}\right)\;\frac{dq_{LM}(t)}{dt}=\mu_{LM}\times q_{LM}(t)$$

$$\frac{1}{N_{LAB}(t)}\times \frac{dN_{LAB}(t)}{dt}={\left(\frac{q_{LAB}(t)}{1+q_{LAB}(t)}\right)\times \mu }_{LAB}\times \left(1-\frac{N_{LM}}{{{Unit}_{Size}\times 10}^{{MPD}_{LM}}}\right)\times \left(1-\frac{N_{LAB}}{{{Unit}_{Size}\times 10}^{{MPD}_{LAB}}}\right)\;\frac{dq_{LAB}(t)}{dt}=\mu_{LAB}\times q_{LAB}(t)$$

setting *N_0 LM_*, *N_0 LAB_*, *q_0 LM_*, and *q_0 LAB_* as the initial values. The outputs of the function are the numbers of *L. monocytogenes* and LAB in the food unit at time *t* (*N_1 LM_*, *N_1 LAB_* in CFU) and the natural logarithm of *q* values at time *t* for *L. monocytogenes* and LAB (*lnq_t LM_*, *lnq_t LAB_*). In the R script, this function is vectorized for all parameters, meaning that it is prepared to receive and return vectors of simulated data. The function was written in C++.

### Appendix A.14. The Function sfCharacteristics

The function sfCharacteristics() samples, at the lot level, the environmental (intrinsic and extrinsic) characteristics of the RTE seafood product, acting therefore as a feeder function for the sfMejlholmDalgaard() and sfMejlholmDalgaardLAB() functions to estimate the specific growth rates of *L. monocytogenes* and LAB, respectively, in the RTE seafood. The sampling of the environmental characteristics of the RTE seafood product assumes that all the units produced in a lot have the same characteristics. Furthermore, since the functions sfMejlholmDalgaard() and sfMejlholmDalgaardLAB() are deterministic, the specific growth rates of *L. monocytogenes* and LAB calculated thereof are the same for all the units produced in a lot.

For every lot *i*, the RTE seafood characteristics are sampled from multiple Pert distributions defined by minimum (*x_min_*), mode (*x_mode_*), and maximum (*x_max_*) values of the given characteristic *x*. The lot-specific characteristics are sampled as follows:

$a_{w\;i}\;\sim \;Pert\;\left(a_{w\;min},\;a_{w\;mode},\;a_{w\;max}\right)$ or ${NaCl}_{i}\;\sim \;Pert\;\left({NaCl}_{min},\;{NaCl}_{mode},{NaCl}_{max}\right)$, ${pH}_{i}\;\sim \;Pert\;\left({pH}_{min},\;{pH}_{mode},{pH}_{max}\right)$, ${LA}_{tot\;i}\;\sim \;Pert\;\left({LA}_{tot\;min},\;{LA}_{tot\;mode},\;{LA}_{tot\;max}\right)$, ${Phe}_{i}\;\sim \;Pert\;\left({Phe}_{min},\;{Phe}_{mode},{Phe}_{max}\right)$, ${nit}_{i}\;\sim \;Pert\;\left({nit}_{min},\;{nit}_{mode},{nit}_{max}\right)$, ${CO2}_{equi\;i}\;\sim \;Pert\;\left({CO2}_{equi\;min},\;{CO2}_{equi\;mode},{CO2}_{equi\;max}\right)$, ${AA}_{tot\;i}\;\sim \;Pert\;\left({AA}_{tot\;min},\;{AA}_{tot\;mode},\;{AA}_{tot\;max}\right)$, ${DA}_{tot\;i}\;\sim \;Pert\;({DA}_{tot\;min},\;{DA}_{tot\;mode},\;{DA}_{tot\;max})$, ${BA}_{tot\;i}\;\sim \;Pert\;({BA}_{tot\;min},\;{BA}_{tot\;mode},\;{BA}_{tot\;max})$, ${CA}_{tot\;i}\;\sim \;Pert\;({CA}_{tot\;min},\;{CA}_{tot\;mode},\;{CA}_{tot\;max})$, and ${SA}_{tot\;i}\;\sim \;Pert\;({SA}_{tot\;min},\;{SA}_{tot\;mode},\;{SA}_{tot\;max})$,

The inputs of the function sfCharacteristics() are the number of lots, *r*, and the Pert parameters defining the environmental characteristics of RTE seafoods. The values of the Pert parameters used are different for smoked fish and gravad fish, and they are compiled in Table 4. The function’s output is a list of vectors of length *r* for *a_w_* (or *NaCl*), *pH*, *LA_tot_*, *Phe*, *nit*, *CO2_equi_*, *AA_tot_*, *DA_tot_*, *BA_tot_*, *CA_tot_*, and *SA_tot_*.

### Appendix A.15. The Function sfColdChain

The inputs of sfColdChain() are the inputs and parameters of all auxiliary functions (sfMejlholmDalgaard(), sfMejlholmDalgaardLAB(), sfGrowthJameson(), and sfCharacteristics()), whose values are passed, accordingly. Although the cardinal parameters of all environmental factors are pre-established as default in the functions sfMejlholmDalgaard() and sfMejlholmDalgaardLAB() for both *L. monocytogenes* and LAB, respectively, it is still possible to alter any of them by providing the function sfColdChain() with the updated parameter(s).

The function sfCharacteristics() is first applied to sample the lot-specific environmental characteristics of the RTE seafood product for all lots *r*,

$$\left\{{NaCl}_{i},{pH}_{i},{LA}_{tot\;i},{Phe}_{i},{nit}_{i},{CO2}_{equi\;i},{AA}_{tot\;i},{DA}_{tot\;i},{BA}_{tot\;i},{CA}_{tot\;i},{SA}_{tot\;i}\right\}=sfCharacteristics\;\left(r,\;{NaCl}_{min},{NaCl}_{mode},{NaCl}_{max},{pH}_{min},{{pH}_{mode},{pH}_{max},LA}_{tot\;min},{{LA}_{tot\;mode},\;{LA}_{tot\;max},\;Phe}_{min},{Phe}_{mode},\ldots \right.\;{nit}_{min},{{nit}_{mode},\;{nit}_{max},\;CO2}_{equi\;min},\;{CO2}_{equi\;mode},\;{CO2}_{equi\;max},{AA}_{tot\;min},{{AA}_{tot\;mode},\;{AA}_{tot\;max},\;DA}_{tot\;min}\ldots \;\left.{{DA}_{tot\;mode},\;DA}_{tot\;max},{{BA}_{tot\;min},\;{BA}_{tot\;mode},\;BA}_{tot\;max},{CA}_{tot\;min},{CA}_{tot\;mode},{CA}_{tot\;max},{{SA}_{tot\;min},\;{SA}_{tot\;mode},\;SA}_{tot\;max}\right)\;$$

Then, the initial physiological state parameter for *L. monocytogenes*, *h_0 LM i_*, is sampled for every lot *i* from a normal distribution with mean *μ_h0_* = 2.8 and standard deviation *σ_h0_* = 4.6 (assumed from Couvert et al. [71]; Table 4) and subsequently converted to *q_0 LM i_* to be used as a parameter of the Baranyi–Roberts growth model.

$$h_{0\;LM\;i}\;\sim \;Normal\;\left(\mu_{h0},\;\sigma_{h0}\right)\;h_{0\;LM\;i}>0;\;i=1,\;2,\;\ldots ,\;r$$

$$q_{0\;LM\;i}=log\left(\frac{1}{exp\left(h_{0\;LM\;i}\right)}-1\right)$$

The log-transformed parameter related to the initial physiological state of LAB cells (ln *q_0 LAB i_*) is sampled from a Pert distribution, whose parameters *ln q_0 LABmin_* = −12*, ln q_0 LABmode_* = 2.73, and *ln q_0 LABmax_* = 1.26 were estimated from Couvert et al. [71] (Table 4). Thus, for every lot *i*, *q_0 LAB i_* is obtained as

$$q_{0\;LAB\;i}\;\sim \;exp\left\{Pert\;\left({\ln q}_{0\;LABmin},\;{\ln q}_{0\;LABmode},\;{\ln q}_{0\;LABmax}\right)\right\}\;i=1,\;2,\;\ldots ,\;r$$

The maximum population density of *L. monocytogenes* (*MPD_LM_*) and that of LAB *MPD_LAB_*) in RTE seafood are sampled for every lot *i* from Pert distributions, whose parameters *MPD_LM min_* (6.60 log_10_ CFU/g), *MPD_LM mode_* (7.36 log_10_ CFU/g), *MPD_LM max_* (8.20 log_10_ CFU/g), *MPD_LAB min_* (8.0 log_10_ CFU/g), *MPD_LAB mode_* (8.5 log_10_ CFU/g), and *MPD_LAB max_* (9.0 log_10_ CFU/g) were obtained from Pérez-Rodríguez et al. [24] and Mejlholm and Dalgaard [54], respectively (Table 4).

$${MPD}_{LM\;i}\;\sim \;Pert\;\left({MPD}_{LM\;min},{MPD}_{LM\;mode},{MPD}_{LM\;max}\right)\;i=1,\;2,\;\ldots ,\;r$$

$${MPD}_{LAB\;i}\;\sim \;Pert\;\left({MPD}_{LAB\;min},{MPD}_{LAB\;mode},{MPD}_{LAB\;max}\right)\;i=1,\;2,\;\ldots ,\;r$$

The mean concentration of LAB in RTE seafood after packaging (${\bar{C}}_{0\;LAB\;i}$, log_10_ CFU/g) is sampled for every lot *i* from a Pert distribution with the parameters of minimum (${\bar{C}}_{0\;LAB\;min}$), mode (${\bar{C}}_{0\;LAB\;mode}$), and maximum (${\bar{C}}_{0\;LAB\;max}$). These values were assumed the same for smoked fish and gravad fish and are compiled in Table 4.

$${\bar{C}}_{0\;LAB\;i}\;\sim \;Pert\;\left({\bar{C}}_{0\;LAB\;min},{\bar{C}}_{0\;LAB\;mode},{\bar{C}}_{0\;LAB\;max}\right)\;i=1,\;2,\;\ldots ,\;r$$

If information is provided on the within-lot variability in the concentration of LAB (within-lot standard deviation, σ_C0 LAB wl_), the numbers of LAB in the RTE seafood units (*N_0 LAB_*) are estimated as

$$N_{0\;LAB\;ij}\;\sim \;10^{Normal\;\left({\bar{C}}_{0\;LAB\;i},\;{\;\sigma }_{C0\;LAB\;wl}\right)}\;i=1,\;2,\;\ldots ,\;r;\;j=1,\;2,\;\ldots ,{\;c}_{p}$$

In the present QRA model, ${\;\sigma }_{C0\;LAB\;wl}$ was set to zero. Next, the lot-specific cold chain time (*time_cc_*) and temperature (*Temp_cc_*) are sampled. If the correlation between time and temperature during the cold chain (*CorTimeTemp_cc_*) is different from zero, *time_cc_* and *Temp_cc_* are sampled for every lot *i* from their respective Pert distributions, targeting the rank correlation value of *CorTimeTemp_cc_* (function cornode from the mc2d package; [78]). Else, *time_cc_* and *Temp_cc_* are independently sampled.

$${time}_{cc\;i}\;\sim \;Pert\;\left({time}_{cc\;min},{time}_{cc\;mode},{time}_{cc\;max}\right)\;i=1,\;2,\;\ldots ,\;r\;{Temp}_{cc\;i}\;\sim \;Pert\;\left({Temp}_{cc\;min},{Temp}_{cc\;mode},{Temp}_{cc\;max}\right)\;i=1,\;2,\;\ldots ,\;r$$

At this point, the cold chain auxiliary microbial kinetic functions can be used. The growth rates *of L. monocytogenes* (*μ_LM i_*) and LAB (*μ_LAB i_*) in RTE seafood for every lot *i* are computed by the functions

$$\mu_{LM\;i}=sfMejlholmDalgaard\left({Temp}_{cc\;i},\;{NaCl}_{i},\;{pH}_{i},{LA}_{tot\;i},{Phe}_{i},{nit}_{i},{CO2}_{equi\;i},{AA}_{tot\;i},{DA}_{tot\;i},{BA}_{tot\;i},{CA}_{tot\;i},{SA}_{tot\;i}\;\right)\;i=1,\;2,\;\ldots ,r$$

$$\mu_{LAB\;i}=sfMejlholmDalgaardLAB\left({Temp}_{cc\;i},\;{NaCl}_{i},\;{pH}_{i},{LA}_{tot\;i},{Phe}_{i},{nit}_{i},{CO2}_{equi\;i},{AA}_{tot\;i},{DA}_{tot\;i},{BA}_{tot\;i},{CA}_{tot\;i},{SA}_{tot\;i}\;\right)\;i=1,\;2,\;\ldots ,r$$

Finally, the numbers of *L. monocytogenes* and LAB in the RTE seafood units after *time_CC_* (*N_cc LM_*, *N_cc LAB_* in CFU) and the natural logarithm of *q* values at time *t* for *L. monocytogenes* and LAB (*lnq_cc LM_*, *lnq_cc LAB_*) are obtained using the function sfGrowthJameson(),

$$\left\{N_{cc\;LM\;ij},\;N_{cc\;LAB\;ij},\;{\ln q}_{cc\;LM\;ij},{\ln q}_{cc\;LAB\;ij}\;\right\}\;=sfGrowthJameson\left(N_{Pack\;ij}\;,\;{N_{0\;LAB\;ij},\;time}_{cc\;i},\;q_{0\;LM\;i},\;q_{0\;LAB\;i},\;\mu_{LM\;i},\;\mu_{LAB\;i},\;{MPD}_{LM\;i},{MPD}_{LAB\;i},\gamma ,{Unit}_{SizePack}\;\right)\;i=1,\;2,\;\ldots ,\;r;\;j=1,\;2\ldots ,{\;c}_{p}$$

The lot-specific vectors *MPD_LM_* and *MPD_LAB_* are also returned.

### Appendix A.16. The Function sfPortioning

The number of *L. monocytogenes* (*N_Portion ij_*, [CFU]) in a portion of RTE seafood (*Serv_size_* [g]) taken from pack *j* produced in lot *i* is sampled from a Beta-binomial distribution,

$$N_{Portion\;ij}\;\sim \;Binomial\left(N_{Home\;LM\;ij},\;Beta\left(b,b\left(n_{serv}-1\right)\;\right)\right)$$

where *b* is the dispersion factor of the Beta distribution and *n_serv_* is the rounded number of servings from a pack of RTE seafoods,

$$n_{serv}=\left\lceil \left.\frac{{Unit}_{SizePack}}{{Serv}_{size}}\right\rceil \right.$$

The inputs of the function are the *L. monocytogenes* load in the RTE seafood pack unit at the time of consumption, *N_Home LM_*, the net weight of the pack, *Unit_SizePack_*, and the serving size, *Serv_size_* (which was set to be equal to one slice, *w_Slice_* = 32.5 g), and the output is the contamination matrix *N_Portion_*, containing the dose of *L. monocytogenes* in a serving. In addition, the function sfPortioning() returns the mean prevalence of contaminated lots (*P_Tested_*, a scalar) and the lot-specific probabilities of contaminated lots (*Prob_UnitPos Tested_*, a vector) unchanged.
